# Known phyla dominate the Tara Oceans RNA virome

**DOI:** 10.1093/ve/vead063

**Published:** 2023-11-08

**Authors:** Robert Edgar

**Keywords:** Taxonomy polymerase, RNA viruses, metagenomics, evolution, phylogeny, RNA-dependent RNA polymerase, HMM-based homology detection, taxonomy

## Abstract

A recent study proposed five new RNA virus phyla, two of which, ‘Taraviricota’ and ‘Arctiviricota’, were stated to be ‘dominant in the oceans’. However, the study’s assignments classify 28,353 putative RdRp-containing contigs to known phyla but only 886 (2.8%) to the five proposed new phyla combined. I re-mapped the reads to the contigs, finding that known phyla also account for a large majority (93.8%) of reads according to the study’s classifications, and that contigs originally assigned to ‘Arctiviricota’ accounted for only a tiny fraction (0.01%) of reads from Arctic Ocean samples. Performing my own virus identification and classifications, I found that 99.95 per cent of reads could be assigned to known phyla. The most abundant species was Beihai picorna-like virus 34 (15% of reads), and the most abundant order-like cluster was classified as *Picornavirales* (45% of reads). Sequences in the claimed new phylum ‘Pomiviricota’ were placed inside a phylogenetic tree for established order *Durnavirales* with 100 per cent confidence. Moreover, two contigs assigned to the proposed phylum ‘Taraviricota’ were found to have high-identity alignments to dinoflagellate proteins, tentatively identifying this group of RdRp-like sequences as deriving from non-viral transcripts. Together, these results comprehensively contradict the claim that new phyla dominate the data.

## Introduction

In 2017, fewer than 5,000 RNA virus species were known (Wolf et al. 2018). Since then, high-throughput metagenomics has dramatically expanded the known RNA virome with studies, such as Yangshan Deep-Water Harbour (YDWH) (Wolf et al. 2020), Serratus (Edgar et al. 2022) and the Tara Oceans project (Zayed et al. 2022), which collectively identified more than 140,000 new species. These new viruses are classified primarily on the basis of their RNA-dependent RNA polymerase (RdRp) gene sequences. RNA virus taxonomy is defined by the International Committee on the Taxonomy of Viruses (ICTV). The upper ranks of realm, kingdom, phylum, and class were recently added to the ICTV taxonomy, primarily on the basis of a predicted phylogeny described in Wolf et al. (2018). The raw data provided by Zayed et al. (2022) (henceforth called Tara) comprises environmental metagenomic sequencing reads for 1,703 RNA-seq runs (BioProject PRJEB402). Reads were assembled, and 44,799 RdRp-containing contigs were identified by hidden Markov model (HMM) search. These contigs were clustered at 90 per cent nucleotide (nt) identity, yielding 5,504 species-like clusters (operational taxonomic units, OTUs). Based on analysis of the data, Tara identified five new RNA virus phyla within the kingdom *Orthornaviria* of the realm *Riboviria*. According to their Abstract, “[s]pecies’-rank abundance determination revealed that viruses of the new phyla “Taraviricota” … and “Arctiviricota” are widespread and dominant in the oceans’. There appears to be no code or intermediate data supporting this claim; in particular, no read mapping results or species abundance tables were provided to the best of my knowledge (for further discussion, see Supplementary Note N1). For the re-analysis reported here, a read mapping and analysis protocol was therefore implemented from scratch. Code and intermediate data supporting this re-analysis are provided in supplementary materials, Zenodo data repositories and github.

## Methods

### Identification of RdRp sequences

RdRp-scan (Charon et al. 2022) HMMs were used to identify RdRp sequences in Tara’s contigs and in GenBank. RdRp-scan was constructed using a similar procedure to Tara’s HMMs. GenBank RdRps were identified by using RdRp-scan to search the NBCI non-redundant protein database (Wheeler et al. 2005), to which RdRp sequences from Wolf et al. (2018) and RdRp-scan hits to NCBI RefSeq genomes for ICTV species were added. To screen for reverse transcriptases which are close homologs of RdRp, PFAM (Finn et al. 2014) models PF00078 and PF07727 were added to the HMM library. The *E*-value threshold was set to 0.001. Operationally, NCBI taxonomy annotations (Schoch et al. 2020) were used as as a reference standard, noting that many of these annotations are tentative assignments from sequence rather than authoritative assignments by ICTV.

### Trimming RdRp sequences to a homologous segment

In general, the boundaries of a viral RdRp gene cannot be identified by sequence methods because RdRp often occurs in multi-gene/multi-domain ORFs together with other genes and domains, and there is no clear sequence signal delineating the transition from one gene to another (see also Supplementary Note N2). Similarly, domains can be identified within RdRp (in particular, the palm domain, which is universally present), but the domain content varies, and domain boundaries are fuzzy. Alignment, clustering, and tree-building ideally requires an approximately globally alignable segment, which is provided by the palmprint region spanning conserved motifs A, B, and C in the palm domain (Babaian and Edgar 2022), see Supplementary Note N2 for further details. The palmprint is *∼*100 amino acids (aa) in length, which is well-suited for clustering into species-like OTUs. For higher classification, longer sequences are preferable, and palmprints were therefore extended by adding up to 150aa flanking sequence on both sides, giving ‘palmcores’ which are ∼400aa segments corresponding roughly to the trimming used in Wolf et al. (2018). The choice of 150aa was a compromise between the conflicting goals of including more palm domain sequence and avoiding overflow into neighbouring domains which are not homologous between distantly related viruses. The A, B, and C motifs were identified using position-specific scoring matrices (PSSMs) in palmscan (Babaian and Edgar 2022). A palmcore was considered to be intact if it had at least 125aa on both flanks; otherwise, it was considered to be a fragment and excluded from classification. Palmcores identified by palmscan from RdRp-scan hits to GenBank and Tara contigs were separately clustered at 90 per cent aa identity using UCLUST (Edgar 2010), giving palmcore species-like OTUs (pc-sOTUs, to distinguish from Tara species-like OTUs, T-sOTUs). Then, pc-sOTUs from GenBank and Tara’s contigs were combined and clustered at 50 per cent aa identity to give palmcore family-like OTUs (pc-fOTUs, to distinguish from T-fOTUs in the Tara paper which were similarly obtained by 50 per cent aa clustering).

### Condensed subtree phylum-like clustering

I have previously shown that the currently defined RNA virus phyla are likely to be substantially polyphyletic, and also that state-of-the-art alignment and tree estimation methods break down when applied to deep RNA virus phylogeny, where high bootstrap values may be artefacts of systematic alignment errors (Edgar 2022). I am not aware of any method which could enable demonstrably accurate placement of novel, highly diverged RdRp sequences in a phylogenetic tree which is deep enough to resolve phyla or phylum-like clades. To obtain phylum-like clusters, a new algorithm, Condensed Subtree Phylum-like Clustering (CSPC), was implemented. CSPC was designed to find a best-fit approximation to the Wolf et al. (2018) tree. A high-diversity ensemble of multiple sequence alignments (MSAs) of pc-fOTUs was constructed by Muscle5 (Edgar 2022). Diversity of the ensemble was increased by randomly selecting a different sequence from each pc-fOTU in each input set, with replacement. For each MSA, a tree was constructed by FastTree (Price, Dehal, and Arkin 2010) with leaves labelled by pc-fOTUs. Each tree was condensed according to GenBank phylum annotations using newick (Edgar 2022) (https://github.com/rcedgar/newick). The condensed tree with maximum phylum monophyly (see Edgar 2022 Fig. S6) was selected (guide tree permutation *abc*, HMM perturbation seed 6), and phylum annotations were assigned to unlabelled leaves according to the best-fit subtree for each phylum. This procedure assigned a phylum to 2,547/2,590 (98.3%) pc-sOTUs. 1,183/1,200 (98.6%) of pc-fOTUs with NCBI taxonomy phylum annotations fell into their corresponding best-fit phylum subtree, providing post-hoc justification that this method is a good approximate fit to the Wolf et al. (2018) tree which is the basis for ICTV phylum and class rank definitions. Phylum assignments by CSPC are given the CS: prefix, e.g. CS:Pisuviricota, to distinguish from a GenBank assignment to an official ICTV taxon which is written without a prefix, e.g. *Pisuviricota*. Assignments by Tara from their megataxon clusters are given the Mega: prefix, e.g. Mega:Pisuviricota.

### Order-like consensus subtrees

**Figure 1.**
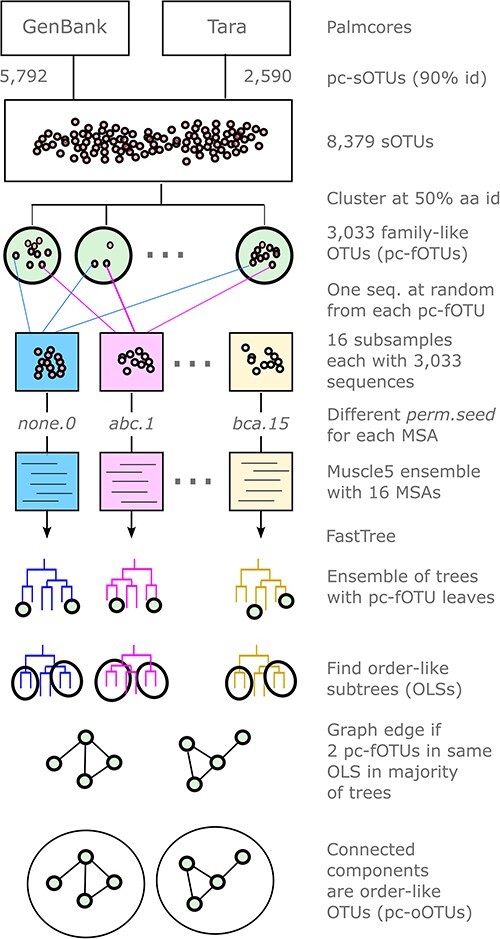
Order-like Consensus Subtrees. The algorithm constructs a diversified ensemble of trees, then selects order-like subtrees (OLS) (Fig. 2) from each tree. Consensus subtrees are identified as connected components in a graph where an edge is drawn if a majority of trees agree that a pair of pc-fOTUs belongs in the same OLS.

**Figure 2.**
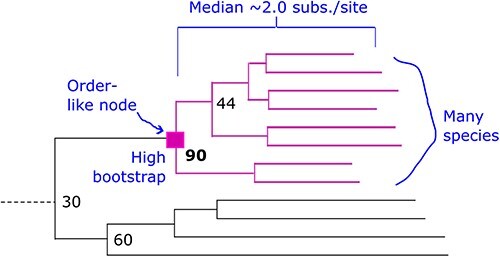
Order-like subtree. The “orderliness” score (Q, see main text) is designed to be larger for nodes which are more likely to be selected as order-like by manual inspection, i.e. have median height around 2 subs./site, have high bootstrap, and have many leaves.

To obtain order-like clusters, I implemented Order-like Consensus Subtrees (OLCS), a new algorithm designed to extract good phylogenetic signal from the noisy trees in an ensemble (Fig. 1). The accuracy of the trees should tend to be high at internal nodes which are at small distances from the leaves (heights), and will tend to degrade with increasing height. Manual inspection of the trees found that the best-fit nodes for orders were at heights of around two substitutions per site (subs./site), but the surrounding topology was quite different in different trees, showing that the topology of a single tree cannot be trusted at this height. The goal of the OLCS algorithm is to extract a more reliable signal from this noise by taking a consensus. For each tree, a disjoint set of order-like internal nodes is identified by greedily selecting nodes which maximise *Q *= **1**(1.5 *≤ h ≤ *2.3 and *n ≥ *8) *B* sqrt(*n*). *Q* is an ‘orderliness’ score designed to be higher for nodes that are more likely to be identified as order-like by manual inspection; i.e. nodes at height around 2 subs./site with many leaves and high bootstrap confidence (Fig. 2). *B* is the bootstrap confidence and *n* is the number of nodes in the subtree. **1** is the so-called indicator function which takes a Boolean condition as an argument, taking value one if the condition is true or zero if the condition is false. Thus, the height of an order-like node is required to be between 1.5 and 2.3 subs./site, and it must contain at least eight leaves. Higher bootstrap is better and more leaves are also better. Taking the square root reduces weighting towards larger subtrees, allowing higher bootstraps to gain importance. Selection proceeds greedily by selecting the internal node with highest *Q*, extracting the order-like subtree (OLS) under this node, repeating until 50 OLSs have been extracted or 95 per cent of the leaves have been extracted. A consensus between OLSs from all trees is obtained by building a graph with an edge for every pair of leaves (i.e. every pair of pc-fOTUs) which are assigned to the same OLS in a majority of trees. A connected component of this graph is then a palmcore order-like OTU (pc-oOTU; there is no comparable cluster type in the Tara paper). Constructing connected components is motivated by transitivity: if species *X* and *Y* belong to the same order, and *Y* and *Z* belong to the same order, then *X, Y*, and *Z* must all belong to the same order. Here, *X* and *Y* are directly connected by an edge if a majority of trees agree they should be in the same order-like group; propagating this inference transitively implies that any two species which are connected by a path through the graph should belong to the same order-like group, and a group defined this way is a connected component. Here, pc-oOTUs are justified empirically by their agreement with taxonomic order annotations in GenBank. Alternatively, pc-oOTUs could be considered to be predictions of monophyletic clades. The accuracy of these predictions could be measured by their ensemble confidences (Edgar 2022), and this procedure could be iterated to improve predicted monophyly. These refinements are deferred to future work. The OLCS algorithm is implemented in the olcs command in newick. The generated pc-oOTUs were numbered consecutively. A pc-oOTU was assigned to the majority GenBank order of its GenBank sequences, if any GenBank sequences are present; otherwise the order is ‘unclassified’. Identifiers for pc-oOTUs are given a OL: (order-like) prefix and an OTU number suffix, e.g. OL:Durnavirales.2, OL:Durnavirales.12 and OL:unclassified.15, to distinguish from a ICTV order which is written without a prefix, e.g. *Durnavirales*. Zero-based numbering is used; e.g. OL:Picornavirales.0 is the first OL: cluster. As these examples show, a single GenBank order may be assigned to two or more pc-oOTUs.

### Read mapping

Reads were mapped using two methods: diamond (Buchfink, Xie, and Huson 2015) and bowtie2 (Langmead and Salzberg 2012). Diamond was used to map reads to all RNA-seq samples from PRJEB402 to pc-sOTUs and T-sOTUs. With bowtie2, both reads and contigs were poly-A-trimmed by bbduk (https://jgi.doe.gov/data-and-tools/software-tools/bbtools/) before mapping; the mapping reference was all nt sequences of the T-sOTU contigs. The bowtie2 pipeline was designed to implement the protocol described in the Tara paper.

### Focused analysis of four geolocations

Four geolocations were selected for further investigation: 72.5°N,44.1°E (Geo-A), 71.6°N,160.9°E (Geo-B), 71.1°N,175.0°E (Geo-C) and 21.1°S,104.8°W (Geo-D) (Supplementary Table S1). Geo-A was selected because Tara’s Fig. 4 shows the largest abundance of ‘Arctiviricota’ at this location, together with a large abundance of ‘Taraviricota’ and should therefore clearly show high abundances of both these phyla by any reasonable abundance metric. Geo-D was selected because it is shown as having one of the highest abundances of ‘Taraviricota’. Geo-B and Geo-C were selected as controls which have no visible abundance of ‘Taraviricota’ and ‘Arctiviricota’ in Tara’s Fig. 4.

### Abundance calculation

Tara’s claims are stated to be based on abundances derived from read mapping, but their abundance measure is not completely described, and the relevant code and abundance summary data are not provided, precluding an attempt to independently replicate their abundance calculations (Supplementary Note N1). Here, reads per kilobase per million total reads (RPKM) (Wagner, Kin, and Lynch 2012; Wolf et al. 2020) is used. If *m* reads map to a contig of length *L* bases in run with *R* reads, RPKM = (1,000 *m/L*)(10^6^*/R*). This corrects for biases due to contig length and the number of reads per run, giving a measure which, hopefully, is commensurate between runs (see Discussion for reasons to be less hopeful). Contigs are trimmed to palmcores with L 1,000nt, so here RPKM ~10^6^m/R. Abundance over multiple samples is obtained by summing RPKM for each sample.

## Results

### Species- and order-like OTUs

**Figure 3.**
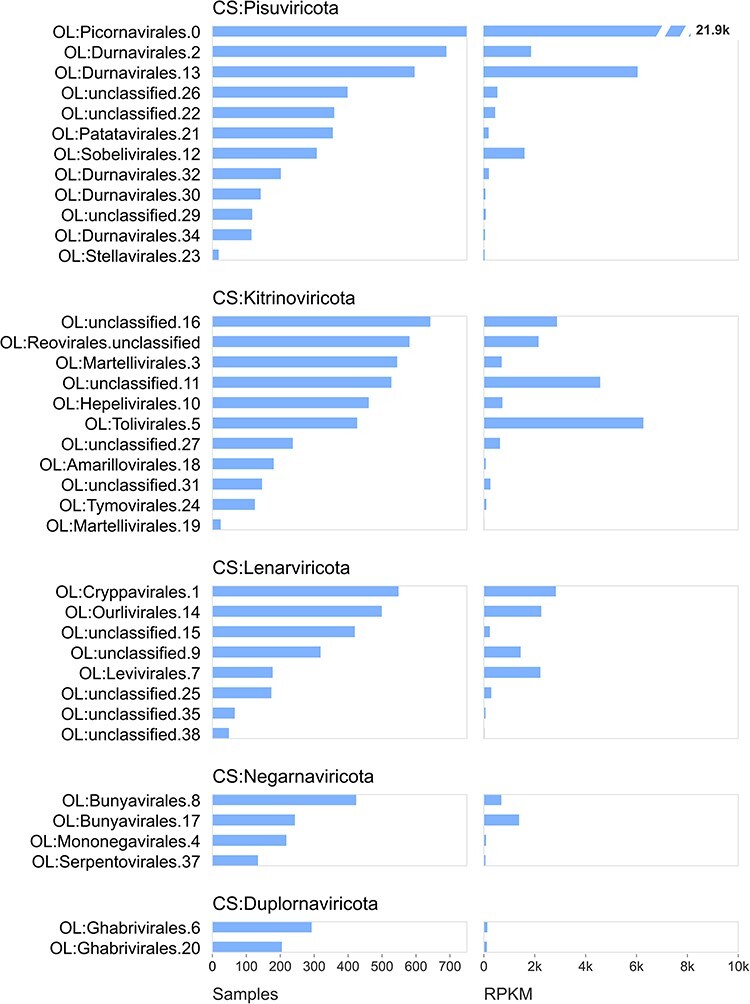
Abundances of order-like OTUs. Histograms show abundances of each pc-oOTU by presence/absence (number of samples) and by RPKM. The most abundant pc-oOTU by both measures is OL:Picrornavirales.0 (note the zero-based numbering) which is assigned to phylum-like cluster CS:Pisuviricota.

Tara’s contigs yielded 2,590 pc-sOTUs which were assigned to all five CS:phyla and to 37 distinct order-like pc-oOTUs, as summarised in Fig. 3; 43/2,590 (1.6%) were not assigned to a CS:phylum. Combined, the pc-sOTUs which were not assigned to a CS:phylum accounted for 3,453 of the 7.45 M mapped reads (0.05%) The most abundant pc-oOTU by RPKM, read count, and sample count was OL.Picornavirales.0, accounting for 3.20 M reads (45%). The top ten pc-sOTUs by RPKM are summarised in Table 1 (expanded in Supplementary Table S2). The most abundant, pc-sOTU.1968, is represented by contig 155SUR1QQSS13_1_k119_109177 which has 92 per cent aa identity to GenBank APG77928.1 and is thereby identified as Beihai picorna-like virus 34, which is assigned to ‘unclassified RNA viruses Shi et al. (2016)’ (NCBI:txid1922348) by the NCBI taxonomy database (Schoch et al. 2020). This pc-sOTU has RPKM 7,808, accounting for 1,134,444 of mapped reads (15%). Seven of the top ten pc-sOTUs are classified to CS:Pisuviricota, four of which are assigned to OL.Picornavirales.0. In addition to pc-sOTU.1968, a further four of the top ten pc-sOTUs also have top BLASTP hits to GenBank proteins classified as Shi et al. (2016) by NCBI. Shi et al. (2016) refers to a study from 2016 (Shi et al. 2016) which reported 1,445 previously unknown RNA virus species associated with barnacles, bivalves, trematodes, sea anemones, shrimp, and other invertebrate aquatic hosts. Submitting the pc-sOTU.1968 representative sequence as a query to PalmID (https://serratus.io/palmid (Babaian and Edgar 2022)) found a 92 per cent aa id match to palmprint u1114 in PalmDB-14 March 2021 (Babaian and Edgar 2022), generating reports and figures showing the geographic distribution and historical record of SRA observations of this virus and its relatives (Fig. 5, Supplementary Table S6). PalmID reported hits to 5,882 SRA runs with submission dates from 2012 through 2021, including 28 runs from Tara’s PRJEB402.

**Table 1.** Top ten most abundant pc-sOTUs by RPKM with classifications. pc-sOTUs are shown in order of decreasing RPKM with classifications by PLCS and OLS, Tara’s megaclusters and BLASTP top hit with NCBI taxonomy. See expanded version in Supp. Table S2 for contig identifiers and RPKM values.

Edgar classification			Tara classification	BLASTP top hit		
EsOTU	CS:Phylum	OL:Order	Mega	Protein	%id	Species
1968	Pisuviricota	Picornavirales.0	Pisuviricota	APG77928.1	92.2	Beihai picorna-like virus 34
932	Kitrinoviricota	Tolivirales.5	Kitrinoviricota	YP_009342302.1	63.9	Wenzhou tombus-like virus 9
1269	Pisuviricota	Picornavirales.0	Pisuviricota	URG14404.1	58.8	Picornaviridae sp.
1057	Pisuviricota	Picornavirales.0	Pisuviricota	URG14456.1	40.4	Picornaviridae sp.
189	Pisuviricota	Picornavirales.0	Pisuviricota	ASM94053.1	97.1	Barns Ness breadcrumb sponge aquatic picorna-like virus 2
355	Pisuviricota	Sobelivirales.12	Pisuviricota	YP_009330048.1	35.6	Hubei sobemo-like virus 3
1008	Lenarviricota	Levivirales.7	Lenarviricota Allassoviricetes	APG76997.1	60.7	Beihai levi-like virus 22
703	Pisuviricota	Durnavirales.2	Pisuviricota	UDL14342.1	83.3	Partitiviridae sp.
1834	Kitrinoviricota	unclassified.16	Wei-like	ASM94040.1	74.4	Barns Ness breadcrumb sponge weivirus-like virus 9
902	Pisuviricota	Durnavirales.13	Pisuviricota	USE08162.1	46.3	Picobirnavirus sp.

### Species-like OTUs from palmprints

Clustering palmprints at 90 per cent aa identity yielded 4,100 palmprint species-like OTUs, 1.6× the number of pc-sOTUs derived from palmcores. This increase reflects that palmprints are shorter (∼100aa vs. ∼400aa), and are therefore more likely to remain intact in fragmented contigs. If a species is represented by a single contig, which is typical, and this contig has an intact palmprint but a fragmented palmcore, then the species is missing from the pc-OTUs but present in the pp-OTUs.

### Community structure

The distribution of pc-sOTU abundances was found to be log-normal-like (Supplementary Fig. S1), consistent with a similar observation by YDWH (Wolf et al. 2020, Extended Data Fig. 10a). Mean species richness per geolocation is 311.5, increasing to 344.2 using the Chao-1 estimator (Chao 1984) of total richness including unobserved species (Supplementary Fig. S2). Median Simpson index (Simpson 1949) is 0.101 (Supplementary Fig. S2), indicating several abundant species rather than dominance by a single species. Projecting species abundance vectors per geolocation onto two dimensions by UMAP (McInnes, Healy, and Melville 2018) resulted in a plot (Supplementary Fig. S3) with no evident natural clusters, suggesting that communities are not readily stratified into environmental or geographic categories, such as arctic/equatorial.

### Number of pc-sOTUs and T-sOTUs

**Figure 4.**
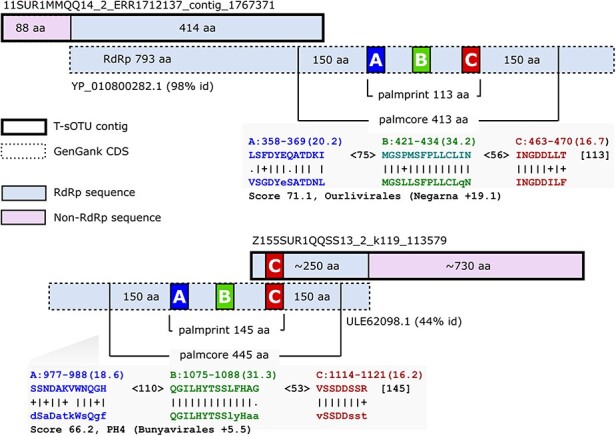
Examples of fragmented Tara species-like representatives. The figure shows two T-sOTU representative contigs aligned to GenBank CDS. Both have < 50% complete RdRp and are missing all (top) or most (bottom) of the palmprint such that these contigs contain non-overlapping fragments of RdRp. This fragmentation, combined with inclusion of fast-evolving, non-RdRp sequence, inflates the number of T-sOTUs compared with pc-sOTUs because pc-sOTUs are palmcores which are approximately globally alignable across all RdRps. Palmcores classify fewer viruses at species-like rank, but are better suited to classification at higher ranks by enabling more robust multiple alignment and tree-building.

Tara reported 5,504 T-sOTUs, 2.1*×* more than the 2,590 pc-sOTUs reported here. The increase is mostly explained by Tara’s use of contig nucleotide identity for clustering, allowing fragmentary RdRp together with non-RdRp sequence (Fig. 4), while this work uses amino acid identity of globally alignable intact RdRp palmcores. According to Tara’s supplementary Table S6, 815 of T-sOTUs have <50 per cent RdRp domain completeness, of which 87 are <20 per cent complete with a minimum of 5 per cent (131DCM1GGZZ14_1). These fragmentary RdRps are discarded from pc-sOTU analysis because they have incomplete palmcores (see examples in Fig. 4). Even if intact RdRps are present, including non-RdRp sequence tends to increase the number of clusters at a given identity threshold because other genes diverge more quickly.

**Figure 5.**
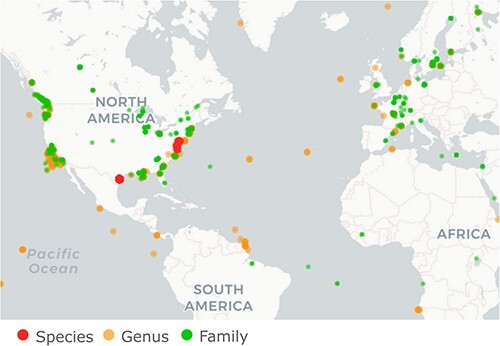
PalmID search for pc-sOTU.1968. This is the most abundant pc-sOTU, identified as Beihai picorna-like virus 34. As seen in this map generated by the PalmID web site, this virus is well-represented in the SRA with hits to 5,882 SRA runs with submission dates from 2012 through 2021, including 28 runs from Tara’s PRJEB402. Most are metagenomes annotated as water-related, including: marine sediment (862 runs), marine (718), aquatic (715), freshwater (546), sediment (461), salt marsh (408), estuary (372), and seawater (107) (Supplementary Table S6).

### T-sOTUs for proposed novel phyla

**Figure 6.**
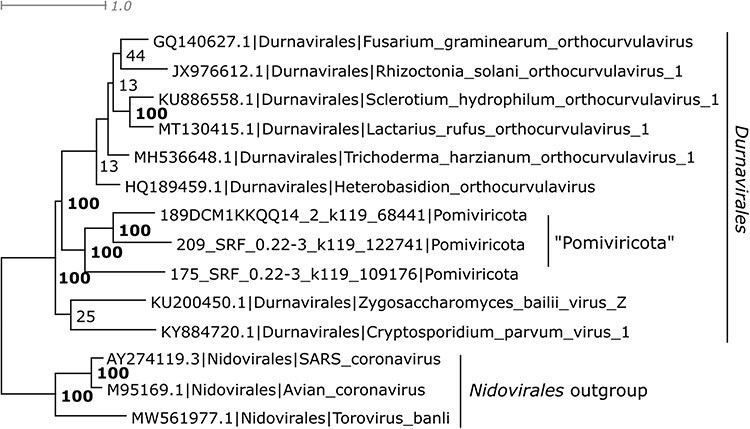
Placement of “Pomiviricota” in Durnavirales. This tree is a consensus of an ensemble generated from Muscle5 alignments with tree estimation by FastTree and RaxML, using Nidovirales as an outgroup to root the tree. Ensemble confidence values are shown for internal tree nodes. “Pomiviricota” is placed inside Durnavirales with 100% confidence.

Tara assigned 120 of their T-sOTUs (2.2%) to their proposed novel phyla. Of these, 14 matched an pc-sOTU at >80 per cent aa identity covering >80 per cent of the pc-sOTU palmcore sequence, as shown in Table 2; the remaining 106 did not match a pc-sOTU. 12/14 of the matching pc-sOTUs are classified to CS:Pisuviricota, four of which were classified as novel phylum ‘Pomiviricota’ by Tara and OL:Durnavirales.32 by me. To confirm the tentative prediction that ‘Pomiviricota’ are *Durnavirales*, an ensemble of Muscle5 palmcore MSAs was generated from the corresponding pc-sOTUs combined with selected ICTV type strains in *Durnavirales*, using three *Nidovirales* strains as an outgroup. Trees were predicted using FastTree and RaxML (Stamatakis 2014). As seen in Fig. 6, the ‘Pomiviricota’ sequences placed inside *Durnavirales* in all trees, giving 100 per cent ensemble confidence for the assignment.

**Table 2.** Representative contigs for proposed novel phyla. This table shows correspondences between the 14 of 120 T-sOTUs for proposed novel phyla which matched pc-sOTUs at > 80% identity over > 80% of the pc-sOTU palmcore. With one exception, they are all assigned to known phyla by PLCS. All “Pomiviricota” sequences are assigned to OL:Durnavirales.32; this tentative assignment to Durnavirales is confirmed by a maximum-likelihood tree ensemble (Fig. 6).

ZsOtu contig	EsOTU contig	Mega	CS:phylum	OL:order
136SUR1SSUU14_2_ERR1712240_contig_794785	136SUR1SSUU14_2_ERR1712240_contig_794785	Taraviricota	Pisuviricota	Unclassified
4DCM1GGMM14_1_ERR1719507_contig_273597	136SUR1SSUU14_2_ERR1712240_contig_794785	Taraviricota	Pisuviricota	Unclassified
81SUR1QQSS14_2_ERR1739838_contig_432813	136SUR1SSUU14_2_ERR1712240_contig_794785	Taraviricota	Pisuviricota	Unclassified
109DCM01QQSS14_1_ERR1719147_contig_151530	136SUR1SSUU14_2_ERR1712240_contig_794785	Taraviricota	Pisuviricota	Unclassified
111SUR1QQSS14_1_ERR1711992_contig_122797	136SUR1SSUU14_2_ERR1712240_contig_794785	Taraviricota	Pisuviricota	Unclassified
145SUR1QQSS14_1_ERR1712051_contig_1080844	136SUR1SSUU14_2_ERR1712240_contig_794785	Taraviricota	Pisuviricota	Unclassified
153MXL01SSUU14_1_ERR1719500_contig_348848	136SUR1SSUU14_2_ERR1712240_contig_794785	Taraviricota	Pisuviricota	Unclassified
144SUR2MMQQ14_1_ERR1711915_contig_93222	136SUR1SSUU14_2_ERR1712240_contig_794785	Taraviricota	Pisuviricota	Unclassified
189_DCM_0.22-3_k119_604244	189DCM1KKQQ14_2_k119_68441	Pomiviricota	Pisuviricota	OL:Durnavirales.32
082_DCM_0.22-3_k119_166667	189DCM1KKQQ14_2_k119_68441	Pomiviricota	Pisuviricota	OL:Durnavirales.32
175_SRF_0.22-3_k119_109176	175_SRF_0.22-3_k119_109176	Pomiviricota	Pisuviricota	OL:Durnavirales.32
209_SRF_0.22-3_k119_122741	209_SRF_0.22-3_k119_122741	Pomiviricota	Pisuviricota	OL:Durnavirales.32
122SUR1MMQQ14_2_ERR1711933_contig_1435227	122SUR1MMQQ14_2_ERR1711933_contig_1435227	Wamoviricota	Duplornaviricota	Unclassified
206_MES_0.22-3_k119_92546	206_MES_0.22-3_k119_92546	Paraxenoviricota	unclassified	Unclassified

### Numbers of reads mapped to T-sOTUs

**Figure 7.**
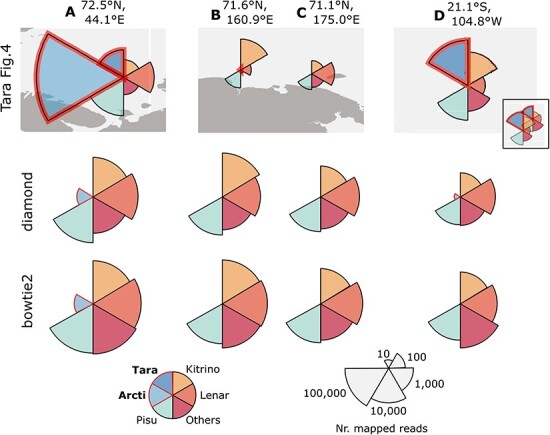
Phylum abundances at four selected geolocations. Pie charts showing abundances according to three different methods: Tara’s Fig. 4 (top), and my implementations using diamond (middle) and bowtie2 (bottom). The pie-chart at location Geo-D in Tara’s Fig. 4 was manually edited to remove the overlapping location adjacent to it (seen in its unedited form in the small inset).Reads are classified into six groups Tara (“Taraviricota”), Kitrino (Mega:Kitrinoviricota), etc., following Tara’s Fig. 4. My pie charts show the number of reads mapped to each group on a logarithmic scale such that the radius of the segment is proportional to log10n where n is the number of reads mapped to the group. The scaling used in Tara’s pie charts is not clearly described, and I therefore did not attempt to reproduce them exactly. However, all methods in this work agree that exactly one read mapped to “Taraviricota” at Geo-A and none at Geo-D, and these results therefore contradict Tara’s figure regardless of their scaling.

**Figure 8.**
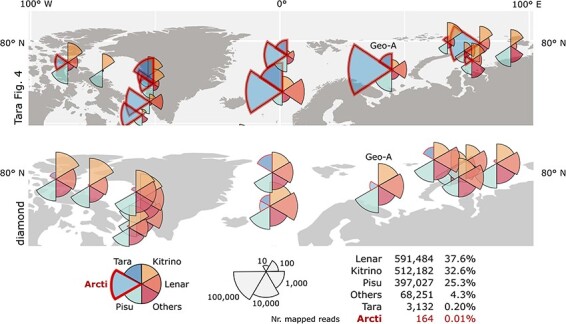
Phylum abundances in the Arctic Ocean. The figure shows abundances in the region bounded by 100°W to 100°E longitude and 60°N to 90°N latitude. Top is this region as depicted in Tara’s Fig. 4. Below is the same region with abundances measured as the numbers of reads mapped by diamond. Reads are classified into six groups Tara (“Taraviricota”), Kitrino (Mega:Kitrinoviricota), etc., following Tara’s Fig. 4. My pie charts show the number of reads mapped to each group on a logarithmic scale such that the radius of the segment is proportional to log10n where n is the number of reads mapped to the group. See caption to Fig. 7 for comment on pie chart scaling. The total numbers of reads mapped in this region is shown at lower right, with only 164 reads assigned to “Arctiviricota” (0.01%). Location Geo-A shows high abundances of both Taraviricota” and “Arctiviricota” in Tara’s Fig. 4; this location is analysed in more detail in Fig. 7.

Table 3 shows total numbers of reads mapped to T-sOTUs across the entire dataset, summarised by Tara’s megataxon assignment. ‘Taraviricota’ and ‘Arctiviricota’ account for 3.75 per cent and 1.28 per cent of the mapped reads, respectively. Mega:Pisuviricota is the most abundant Mega:phylum, with 48.0 per cent of the mapped reads. Table 4 and Fig. 7 shows Mega:phylum abundance by read count mapped to T-sOTUs at locations Geo-A, Geo-B, Geo-C and Geo-D (see Methods). The largest fraction of reads mapped to ‘Arctiviricota’ at Geo-A is 68/445,728 = 0.015 per cent by diamond, and no reads mapped to ‘Taraviricota’ at any of the four geolocations by any of the methods used here. On p.6, third column of their paper, Tara report that ‘vOTUs belonging to the -ssRNA phylum “Arctiviricota” were, on average, the most abundant across most of the Atlantic Arctic waters (Fig. 4).’ Fig. 8 shows a region of the Arctic Ocean where Tara’s Fig. 4 displays the highest abundances for ‘Arctiviricota’. In stark contrast, ‘Arctiviricota’ accounts for only 0.01 per cent of reads mapped by diamond to their T-sOTUs in that region. Note that this analysis used Tara’s species-like OTUs and phylum assignments for read mapping and classification, so the discrepancy cannot be explained by disagreements about classification or by RdRps which were identified by Tara’s analysis but not by mine.

**Table 3.** Numbers of reads mapped by diamond across the complete Tara RNA virome. This table shows the total numbers of reads mapped to each Mega:phylum by diamond for all Tara RNA-seq runs at ≥ 90% aa id. Contig classifications were taken from Tara’s supplementary tables. All contigs classified by Tara were included. This shows that known phyla dominate Tara’s reads according to Tara’s contig classifications.

Mega:phylum	Nr. of mapped reads	Pct. of mapped reads
Pisuviricota	4,948,767	48.0%
Kitrinaviricota	1,880,720	18.3%
Lenarviricota	1,423,964	13.8%
(Undetermined)	1,071,627	10.4%
‘Taraviricota’	385,966	3.75%
Negarnaviricota	349,901	3.40%
‘Arctiviricota’	132,257	1.28%
Duplornaviricota	49,879	0.48%
‘Pomiviricota’	37,470	0.36%
‘Paraxenoviricota’	17,417	0.17%
‘Wamoviricota’	789	0.01%

**Table 4.** Numbers of reads mapped to geolocations A and D. This table shows the number of reads mapped per Mega:phylum by diamond and bowtie2. My results show exactly one read at Geo-A and zero reads at Geo-D mapped to “Taraviricota” according to all methods, contradicting Tara’s Fig. 4 which shows high abundance of “Taraviricota” at both locations. The apparent large discrepancy between diamond and bowtie2 for Mega:Pisuviricota at Geo-D is due to cellular proteins in the full-length contigs which are trimmed in the palmcore mapping reference for diamond (see example in Fig. 9).

	Geo-A		Geo-D	
Mega:phylum	diamond	bowtie2	diamond	bowtie2
Pisuviricota	203,596	508,866	1,736	16,332
Lenarviricota	184,658	418,019	12,420	22,772
Kitrinoviricota	38,186	98,424	622	4,351
Duplornaviricota	5,534	12,394	13	137
Negarnaviricota	3,582	39,060	530	475
‘Pomiviricota’	292	431	62	36
‘Arctiviricota’	68	133	4	0
‘Taraviricota’	1	1	0	0
‘Paraxenoviricota’	0	0	0	0
‘Wamoviricota’	0	0	0	0

### Screening against cellular proteins

**Figure 9.**
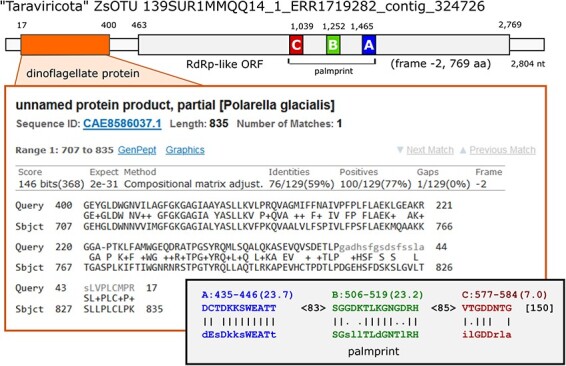
“Taraviricota” species representative with dinoflagellate protein. This T-sOTU contig is 2,804nt. Positions 7 through 400 are aligned to Polarella glacialis protein CAE8586037.1 by BLASTX. Positions 463 through 2,769 are an RdRp-like ORF on the negative strand. Predicted motifs A, B and C according to palmscan are shown in the lower inset. Numbers shown at top are nt coordinates counting from 1 at the beginning of the contig. Predicted motif coordinates by palmscan are aa positions counting from 1 at the start of the ORF. The presence of a cellular protein in this contig suggests that it corresponds to a cellular transcript rather than a virus.

As a check on viral origin, all contigs identified by Tara or me as putatively containing viral RdRp were searched against the NCBI non-redundant protein database using diamond, keeping only top hits with *E *< 10*^−^*^6^. 96 T-sOTUs and 36 pc-sOTUs had a top hit to a protein which is cellular according to its NCBI taxonomy annotation (Supplementary Table S3), suggesting a non-viral origin for the contig. As an example, a ‘Taraviricota’ T-sOTU representative contig with a dinoflagellate protein hit is shown in Fig. 9. As a control, the same search was performed on all *Riboviria* RefSeqs, finding four hits to reverse transcriptases in *Artverviricota* retroviruses, as would be expected, and nine hits to *Orthornavirae* (Supp. Table S4). These nine alignments all have >96 per cent identity to a virus RefSeq genome and are thus identified as viral contaminants which are misannotated as bacterial proteins in GenBank.

## Discussion

### Conflicting analyses

My analysis paints a radically different picture from Tara’s paper, raising the question of how the conflicts can be explained. My ability to investigate this issue was limited by incomplete method descriptions and by missing supporting data and code (Supplementary Note N1).

### Classification of species

RdRp-containing contigs can be classified as known or novel species by sequence alignment, with a 90 per cent identity threshold conventionally considered to be roughly equivalent to species. Identifying known species is not straightforward as GenBank lags behind metagenomics projects such as YDWH (Wolf et al. 2020), Serratus (Edgar et al. 2022) and Tara (Zayed et al. 2022), and RdRp is often not annotated as such (Supplementary Note N2), raising the issue of the unavoidable trade-off between sensitivity and error rate in viral RdRp detection methods such as RdRp-scan which must be applied to known as well as new data. Distinguishing viral RdRp from degraded endogenous virus insertions and close cellular homologs is challenging (Supplementary Note N3), and some rate of false positive errors should therefore be expected, especially for highly-diverged sequences. Assuming that novel viral RdRp sequences have been satisfactorily identified, they can then be clustered at 90 per cent identity to obtain species-like OTUs and thereby quantify the number of novel species. Tara’s use of nucleotide identity and flexible boundaries of cluster representative sequences, allowing fragmentary RdRp genes and the inclusion of non-RdRp flanking sequence, gives roughly double the number of species-like OTUs compared to the use of amino acid identity with globally alignable palmcores in this work. Both approaches are reasonable, and the difference in numbers reflects the essentially arbitrary nature of species definitions and clustering for putative viruses known only from sequence evidence. Using palmprints rather than palmcores enabled the identification of 1.6× more species-like OTUs, illustrating the value of the palmprint as a taxonomic barcode, noting that using palmprints to identify species or species-like OTUs does not preclude requiring additional flanking sequence for other purposes such as classification to higher ranks.

### Classification to higher ranks

Higher ranks are more challenging, as alignment and phylogenetic tree estimation already begins to break down around genus rank (see coronavirus examples in Edgar 2022). Around the rank of order, tree topology is evidently unreliable, requiring elaborate measures to resolve even approximately, as illustrated by the OLCS algorithm introduced here. Sequences may clearly place inside a known genus, family or order, in which case the taxon can be assigned. However, unlike with species, placing outside all named taxa at a higher rank does not necessarily imply that a sequence represents a novel clade because it is generally also possible to extend an existing taxon. An example is provided by the novel clade of coronavirus-like genomes identified by Serratus (Edgar et al. 2022, Fig. 3). My colleagues and I argued that this clade would be best classified as a new genus in *Coronaviridae*, but the tree is also consistent with a new family in *Nidovirales* or a sub-genus of *Deltacoronavirus* by expanding the latter genus. All three options (new family, new genus or new sub-genus) preserve taxon monophyly, and the choice must be made by subjective judgements about classification.

### Classification to phylum

**Figure 10.**
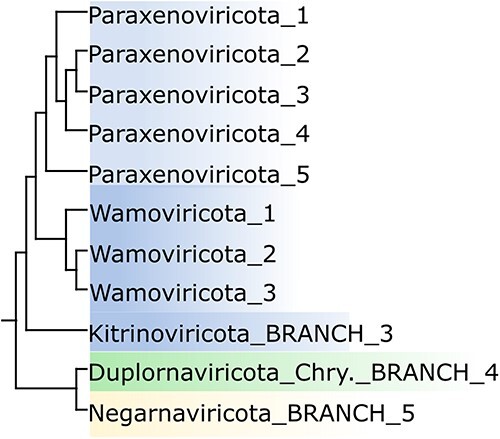
Ambiguous higher taxa from trees. This figure shows a subtree of Tara’s global tree (their Fig. 3A). Assuming this tree is credible and the bootstrap values are informative, then it shows (1) “Paraxenoviricota” and “Wamoviricota” are monophyletic, (2) they are distinct clades, and (3) they are outside Kitrinoviricota. However, it does not show that they should be distinct phyla. It is also consistent with defining “Paraxenoviricota” and “Wamoviricota” as sub-clades of a new phylum, or extending Kitrinoviricota to include both new groups. In the alignment used by Tara to make this tree, Kitrinoviricota is represented by a single sequence, so here it is impossible by construction for any other sequence to place inside this phylum.

RNA virus phyla are currently defined by ICTV accepted proposal 2019.006 G. For example, a virus is assigned to *Pisuviricota* ‘if it has a positive-sense or double-stranded RNA genome and does not infect prokaryotes’ or to *Kitrinoviricota* ‘if it has a positive-sense [RNA genome], does not infect prokaryotes, and does not cluster in RdRp trees with members of *Pisuviricota*’. These specifications invoke genome type and host kingdom, which are not readily inferred from environmental metagenomic sequences. The proposal does not explain how to ‘cluster in RdRp trees’, noting again that state-of-the-art alignment and tree estimation methods fail with highly diverged RdRps (Edgar 2022). The Tara paper does not propose new criteria for phylum identification, or offer any explanation of why the corresponding cryptic sequences should be classified as five new phyla. Tara’s estimated phylogenetic tree exhibits similar ambiguities to the tree for the Serratus coronavirus-like clade, as seen in Fig. 10 here. In this tree, ‘Paraxenoviricota’ and ‘Wamoviricota’ are monophyletic, they are distinct clades, and they are outside *Kitrinoviricota*. However, even if the tree is believed to be correct, these observations would not be sufficient to show that the outlier groups are new or distinct phyla. Alternatively, ‘Paraxenoviricota’ and ‘Wamoviricota’ could be defined as sub-clades of one new phylum, or *Kitrinoviricota* could be extended to cover both. Tara included only one sequence for four of the five ICTV phyla in their top-level alignment and tree, and it is therefore impossible by construction for any other sequence to place within the subtree of an ICTV phylum (with the exception of *Duplornaviricota* which has nine sequences). Thus, their tree cannot support a hypothesis that novel sequences fall outside known phyla.

### Tara’s classifications

**Figure 11.**
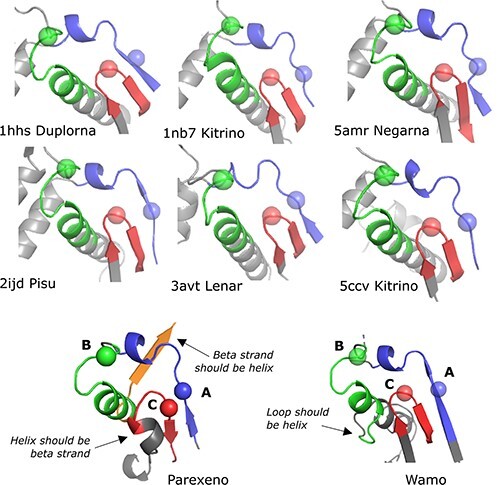
Malformed catalytic cores of “Parexenoviricota” and “Wamoviricota” structures. The figure shows the palmprint in Parexeno and Wamo with six representative solved structures for comparison (phylum names without “viricota” for brevity). The alpha helix of motif B should have five complete turns, but in Parexeno there are three and in Wamo only one. In Parexeno, the three helix turns are followed by a beta sheet which is perpendicular to three helix turns at the corresponding primary sequence positions when aligned to solved structures. In Wamo, the single turn is followed by a loop. In Parexeno, the characteristic antiparallel beta sheet of motif C is replaced by a single-turn helix adjacent to a beta strand.

Tara used MCL (Van Dongen 2008) to create ‘megaclusters’ from pair-wise BLASTP bit scores, identifying each such cluster as a ‘megataxon’. Tara’s paper claims that this method ‘nearly completely recapitulated the previously established phylogeny-based ICTV-accepted taxonomy at the phylum and class ranks (97% agreement)’. This claim is not correct (for details and further discussion see Supplementary Note N4). Tara’s Fig. 3B shows a ‘structure network’ generated by cytoscape (Shannon et al. 2003) which, according to the figure caption, is informative for ‘inferring the early history of orthornavirans’. However, no evidence is provided in support of this remarkable claim, and the protocol for making inferences from this figure is not described (see also Supplementary Note N5). Further, the network is based on predicted structures for new phyla, most of which exhibit truncated and malformed palm domains (see Fig. 11 and Supplementary Note N6). Twelve of the conserved motifs reported by Tara to be present in these structures are in fact absent due to truncation (Supplementary Note N6).

### Can deep RdRp phylogeny be resolved?

Several recent papers have reported methods which approximately reproduced the Wolf et al. (2018) tree, including Tara’s, Neri et al. (2022), and the tree from CSPC reported here. This challenges my recent assertion that deep RdRp phylogeny cannot be accurately resolved (Edgar 2022) because it can be argued that independent methods would not agree with each other unless they are agreeing with a common ground truth. However, similar trees can be obtained by similar systematic biases because all these methods start with some kind of nearest-neighbour clustering. For example, multiple alignment methods such as Muscle v5 use a nearest-neighbour guide tree, but nearest neighbours are not necessarily evolutionary neighbours (Saitou and Nei 1987; Koski and Golding 2001), and alignment bias towards the guide tree can propagate through to a tree estimated from that alignment (Toth et al. 2013; Edgar 2022). There may also be an incentive (i.e. bias) to reproduce current phyla because this step is a prerequisite for claiming new phyla, which may be perceived as a more exciting discovery than a new order or new family when the only observed phenotypes are putative RdRp sequences. Neri et al. (2022) present evidence that all five current ICTV phyla are monophyletic and proceed to identify two outgroups in their tree as new phyla. This evidence can be interpreted differently. Neri et al. (2022) outgroups could be accommodated by extending *Kitrinoviricota*, similarly to Fig. 10. ICTV phyla are not monophyletic because *Duplornaviricota* is paraphyletic with *Negarnaviricota* in the topology accepted by ICTV (proposal 2019.006 G, Fig. 1). Thus, the trees in Wolf et al. (2018) and Neri et al. (2022) disagree not just on branching order, which both papers acknowledge to be uncertain, but also on which major groups are monophyletic. This disagreement supports the conclusion of the Muscle v5 paper (Edgar 2022) that deep RdRp trees cannot be resolved well enough to support robust taxonomic classification at phylum rank. The structure-based tree in Mönttinen, Ravantti, and Poranen (2021) necessarily reflects nearest-neighbour relationships rather than phylogeny because additive distances are required for inferring phylogenies (Felsenstein 2004), and it is not possible to obtain additive distances from structure similarity scores. Therefore, trees derived from structure similarity are not valid predictions of phylogenetic trees. The bias towards grouping nearest neighbours rather than phylogenetic neighbours can also explain how a generic clustering method, such as MCL is able to approximately reproduce branches in the Wolf et al. (2018) tree, but only after an exhaustive search for a clustering with a good fit. Similarly, CSPC relies on generating a variety of trees and choosing (i.e. cherry-picking) one which is close enough to Wolf et al.’s (2018).

### Abundance measure

RPKM was designed to compare expression of different genes from RNA-seq reads by correcting for mRNA/CDS length of the gene and the number of reads per sample (Wagner, Kin, and Lynch 2012). By making these corrections, RPKM enables an inference such as ‘gene *x* was found to be more highly expressed in patient *A* compared to gene *y* in patient *B*’. RPKM was applied to viral RNA-seq metagenomic data by YDWH, using contig length as a proxy for mRNA/genome length. However, gene expression transcriptomics, where each sample is taken from a single individual, is quite different from environmental viral metagenomics, where the biological relevance and interpretation of RPKM is unclear. In a gene expression experiment, a large majority of the reads deriving from host gene transcripts can be identified by mapping reads to the host genome, and the total number of host reads is then appropriate for normalising. In RNA-seq environmental metagenomics such as Tara’s study, there are presumably many diverse hosts, and the ratio of viral to host reads is unknown and presumably highly variable. The number of viral transcripts generated by a single infected cell is also highly variable, even for a given virus strain infecting a given host species—there is higher unevenness between cells in viral gene expression for influenza than wealth inequality of the potential human hosts of an influenza virus living in the USA (Russell, Trapnell, and Bloom 2018). In data such as Tara’s, both host and virus genomes are often unknown, and many reads cannot be assigned to hosts or viruses by mapping. Many virus species, both known and unknown, may be unidentified because read depth was too low to assemble and/or because detection methods such as HMMs are not sufficiently sensitive. Even if all viral reads could be assembled, if a few hundred reads map to a given RdRp-containing contig, the data still cannot say whether these reads were induced by (a) hundreds of transcripts in a single infected host cell, (b) hundreds of infected cells in a single multi-cellular host individual, (c) a few transcripts each from hundreds of hosts, or, more likely, (d) an unresolvable mix of scenarios (a), (b), and (c). Therefore, biologically interesting quantities (numbers of infected hosts, infected cells, virus particles per *µl*, and viral transcripts per cell) cannot be inferred the reads. From these considerations, it seems to me that RPKM is not biologically informative or commensurate between samples. In a large-scale experiment with hundreds of samples, this problem is compounded because read counts across the entire dataset are determined only for the subset of viruses that are assembled in at least one sample, introducing another bias which cannot be detected or corrected, because viruses which are rare in all samples will be missed, while viruses which are rare in most samples and abundant in a few will be detected. This last bias probably substantially skews abundance-based characterisation of community structure (see below). Considering all these limitations and biases, it seems hopeless to obtain biologically informative or commensurate abundances from this type of data, and the biological utility of a proposed abundance metric should therefore be validated, and if that is not possible, then the preferred summary data should be simply presence or absence of detected viruses per sample.

### Community structure

The few thousand species identifiable in the Tara data surely do not tell the whole story. Viruses are believed to represent by far the biggest reservoir of genetic diversity in the oceans, with an estimated *∼*10^30^ genomes causing *∼*10^23^ infections per second (Breitbart et al. 2007). YDWH found a log-normal-like distribution of RPKM abundances, and a similar distribution is reported here. YDWH interpret this distribution as ‘typical in complex environments’, with an excess of high abundances ‘hint[ing at] a dynamic environment producing superabundant viral blooms’. Indeed, many observed species abundance distributions (SADs) are consistent with an approximately log-normal probability density function (Ulrich, Ollik, and Ugland 2010), and by the central limit theorem a community’s SAD will be log-normal if its abundances are determined by many independent random factors (May 1975), giving theoretical grounds to expect that such distributions will be common in practice. However, sampling is often insufficient to observe all rare species, in which case a log-normal distribution will be truncated, hiding many lower-abundance species behind ‘Preston’s veil’ (Dewdney 1998). In the SADs reported here and by YDWH, the low-abundance tail of the bell curve is visible, which would imply that the log-normal is not truncated and hence that most rare species have been sampled. This is apparently confirmed by the Chao-1 estimator of total species richness which is only modestly larger than the observed richness (Supplementary Fig. S2). However, the expectation of vast unexplored virus species richness in the oceans predicts a strongly truncated distribution, and a hypothesis that most species are sampled by the palmcore-derived SAD is contradicted empirically by the finding that there are substantially more species-like OTUs when shorter barcodes are used (palmprints rather than palmcores). This paradox can be resolved by considering the sampling bias described above due to assembling samples individually rather than assembling all reads together. Rare viruses in a given sample will be detected only if they are abundant enough elsewhere to be assembled. Under the natural assumption that most viruses which are rare in one sample are rare or absent in the other samples, it follows that observations of species across the entire dataset will be increasingly depleted with lower abundance. Most likely, the true abundance curve for the complete Tara dataset approximates a truncated log-normal, with truncation where abundances fall below one read per species (Supplementary Fig. S5). This effect may account for the skewed fit to a full (non-truncated) log-normal noted by YDWH; its superficial resemblance to a bell curve arises because sampling bias induces a low-abundance tail. This effect also invalidates the Chao-1 estimator, which assumes that all individuals are equally likely to be observed, while here a given individual from a low-abundance species is less likely to be observed than a given individual from a high-abundance species, if in fact‘individual’viruses in any useful sense can be counted in such data. These examples illustrate how incorrect inferences can arise from uncorrected or unconsidered biases in abundance measures such as RPKM, underscoring that they should be interpreted with caution.

## Conclusion

Tara’s summary data show their proposed novel phyla comprise only a small fraction of their contigs and species-like clusters. My results show that the corresponding contigs account for only a small fraction of their reads, regardless of how they should be classified, and that evidence of viral origin for many of them is equivocal. Together, these results definitively show that the Tara’s proposed new phyla are not ‘dominant in the oceans.’ In fact, known phyla account for a large majority of species and reads, and known phyla thereby dominate the Tara Oceans RNA virome.

## Supplementary Material

vead063_Supp

## Data Availability

Code and data are deposited at: https://github.com/rcedgar/tara_oceans  https://github.com/rcedgar/muscle  https://github.com/rcedgar/newick  https://github.com/rcedgar/palmscan  https://zenodo.org/record/7194888  https://zenodo.org/record/7959728.
