# Supplementary material for: Known phyla dominate the Tara Oceans RNA virome
Source: Virus Evol. 2023 Nov 8;9(2):vead063. doi: 10.1093/ve/vead063 (PMC10649353; doi:10.1093/ve/vead063)
Supplement: vead063_Supp [file vead063_supp.zip › supplementary_figures.docx]

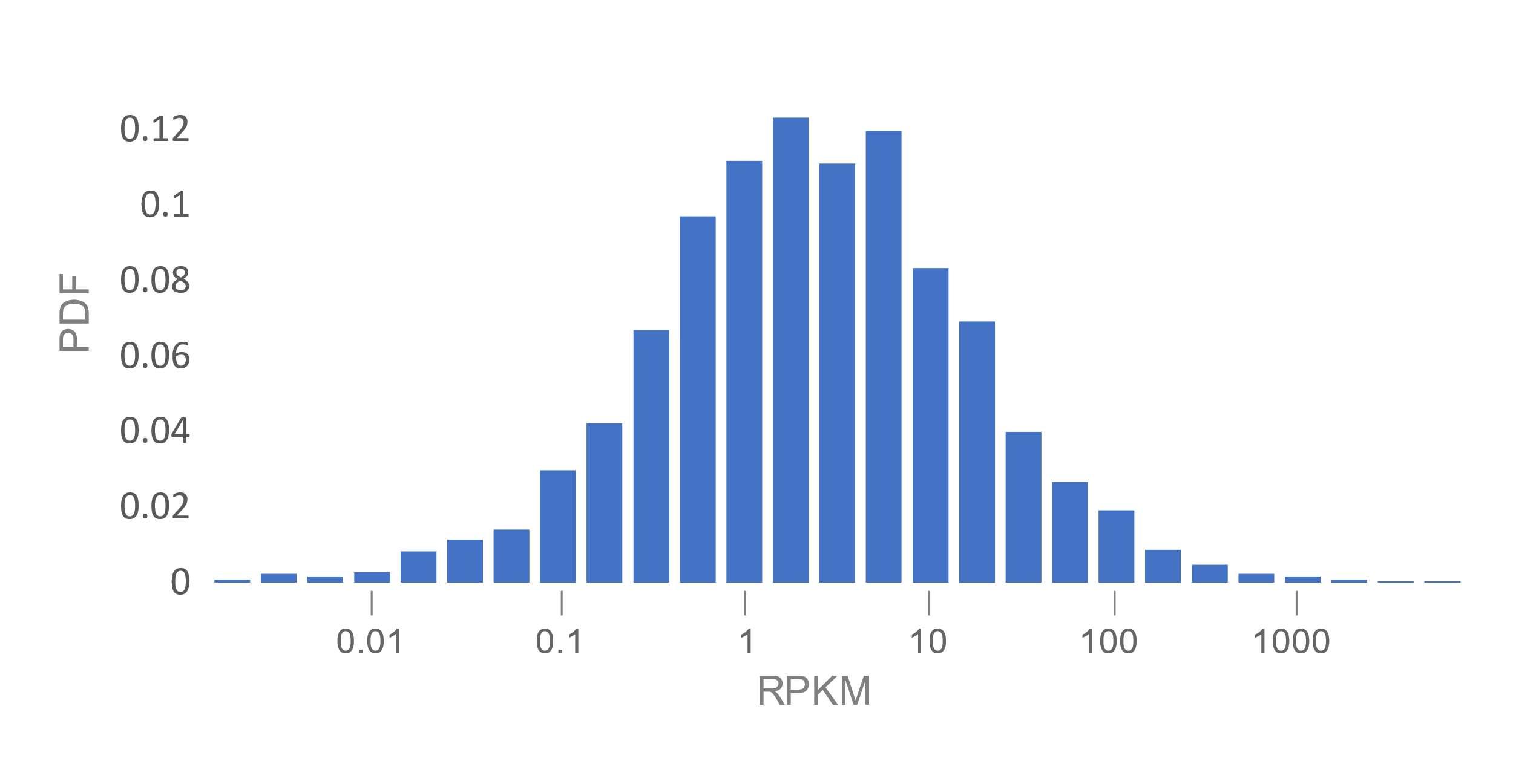
**Figure S1. Abundance distribution of pc-sOTUs by RPKM.**When plotted on a logarithmic axis the distribution has an approximately normal (bell-curve) shape, indicating a log-normal-like probability density function (PDF). See also Fig. S4.


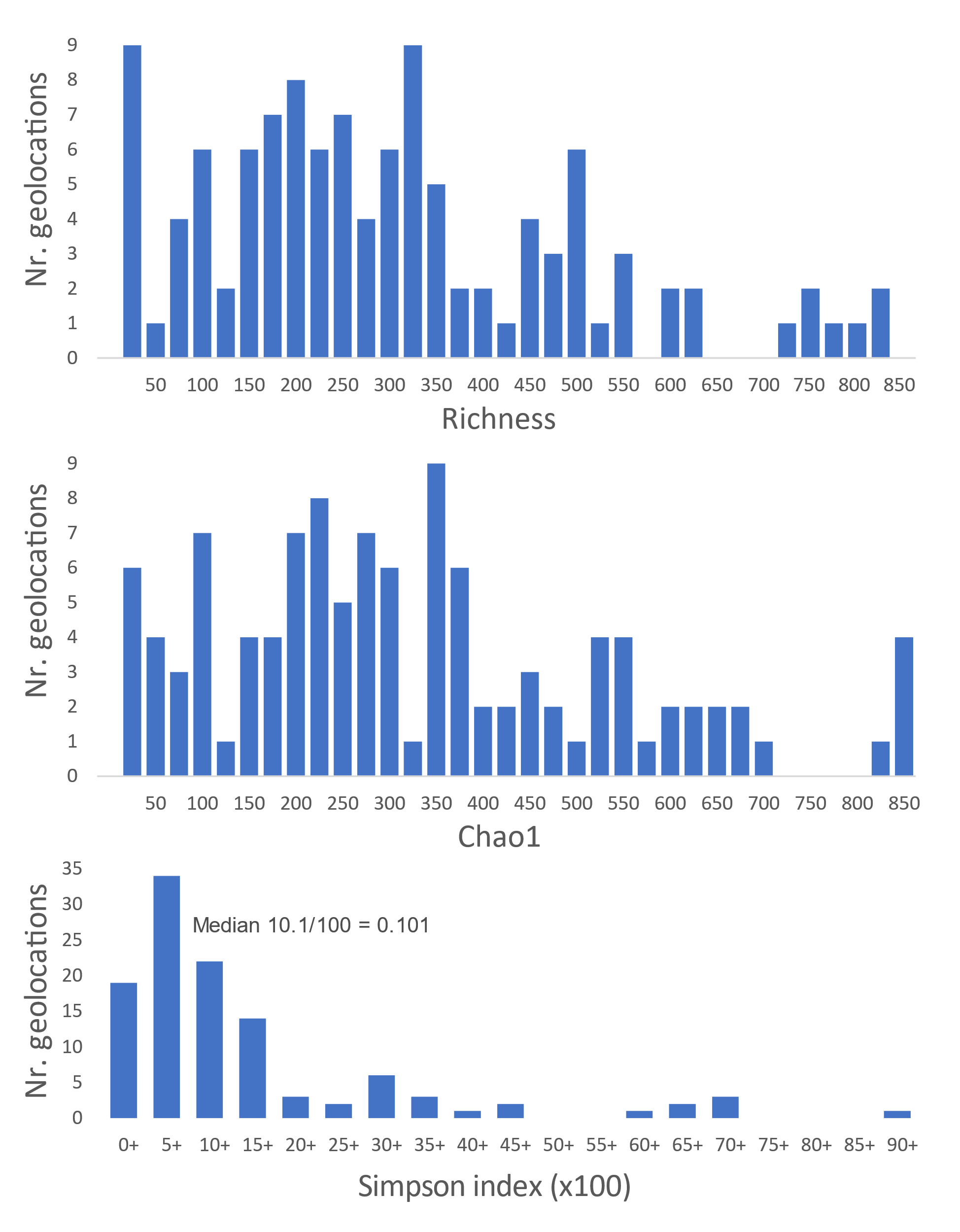


**Figure S2. Alpha diversity metrics for pc-sOTUs**

Richness is the number of distinct pc-sOTUs per sample, i.e. the number of species-like clusters found in the sample. Chao1 is an estimate of the total number of species, including those not observed, by extrapolating the low-abundance tail. Simpson index is the sum over pc-sOTUs of f^2^ where f is the frequency of the OTU. It is the probability that two randomly selected reads will belong to the same OTU. A value close to 1 indicates that a single large OTU dominates the sample, small values indicate that the reads are distributed over many OTUs.


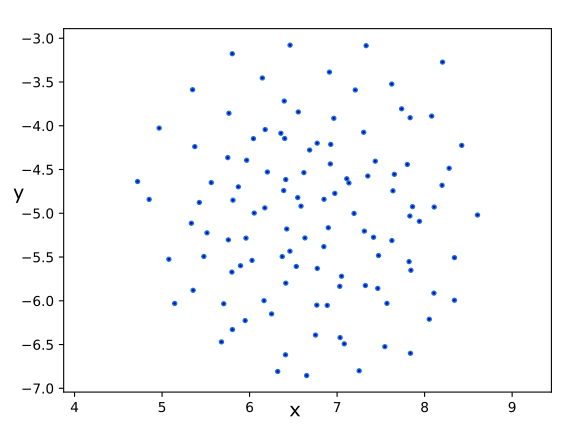


**Figure S3. UMAP projection of pc-sOTU abundance vectors.**The scatter appears random, indicating that the species composition of the samples does not cluster into natural groups such as equatorial / arctic.


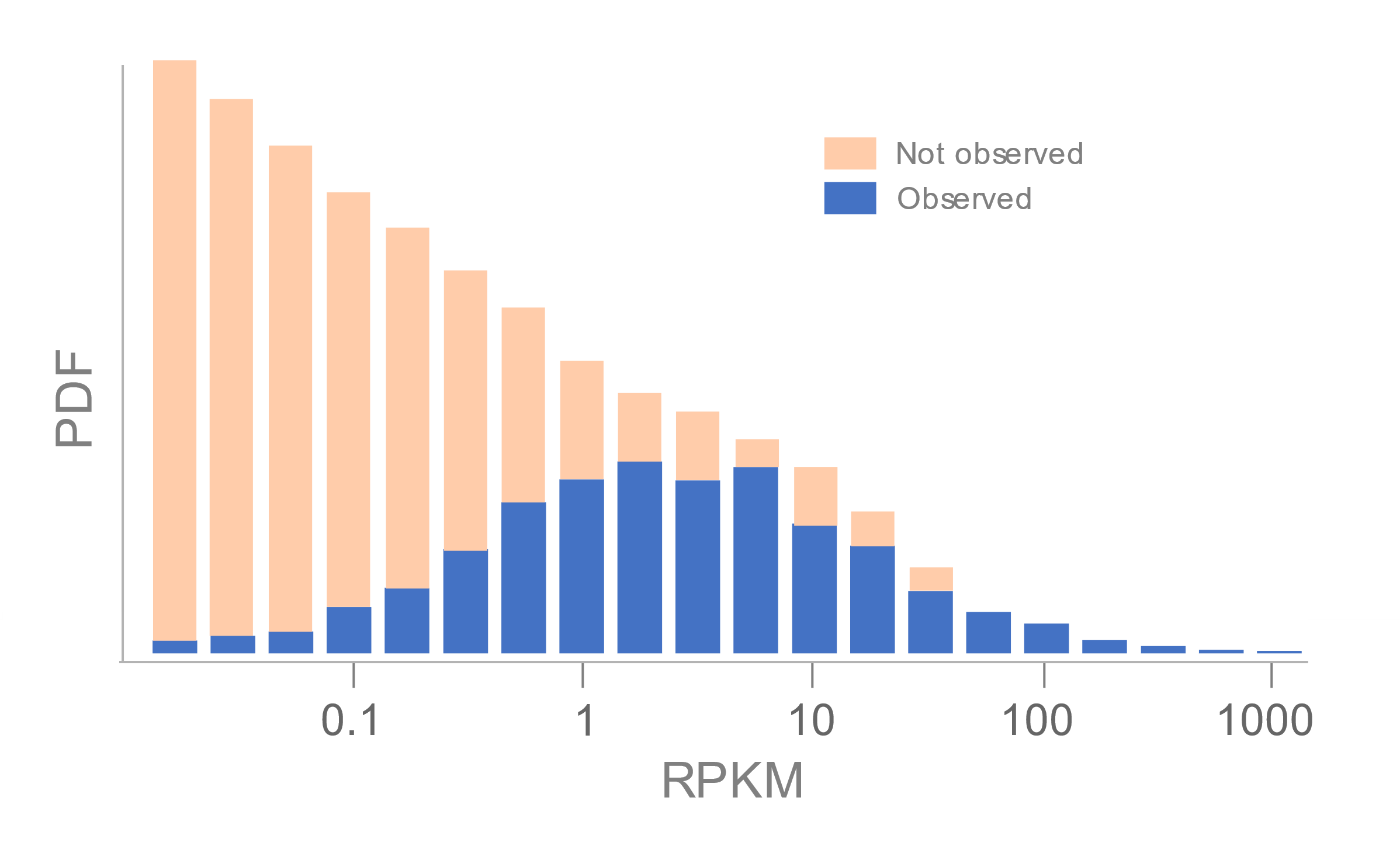


**Figure S4. Suggested truncated log-normal species abundance distribution.**

There is likely to be a bias against observing low-abundance species because a species must be assembled at least once to be observed. Probably, most low-abundance species have low or zero abundance in all samples and are therefore not assembled. I suggest that the underlying distribution is approximately a truncated log-normal with peak far to the left of the observed peak. The *y* axis is Preston's veil line; to the left of the *y* axis are species which do not appear in the data (zero reads). The hypothetical abundances of species which are present in the data but not assembled are shown in orange. The numbers of unobserved species (heights of the orange bars) depend on the mean of the log-normal, which cannot be estimated from this data.
