# Supplementary material for: Known phyla dominate the Tara Oceans RNA virome
Source: Virus Evol. 2023 Nov 8;9(2):vead063. doi: 10.1093/ve/vead063 (PMC10649353; doi:10.1093/ve/vead063)
Supplement: vead063_Supp [file vead063_supp.zip › Supplementary_Note_N1_Missing_supporting_data_and_code.pdf]

Known phyla dominate the Tara Oceans RNA virome

Robert C. Edgar

Supplementary Note N1: Missing supporting methods, data and code.

Claim of "abundant" and "dominant" viruses

The title of Zayed2022 describes "cryptic" (presumably meaning not well-characterized) marine viruses as "abundant", which implies a notably high abundance given its appearance in a short title. The Abstract claims more specifically that "the new phyla "Taraviricota"... and "Arctiviricota" are "dominant in the oceans" by "'[s]pecies'-rank abundance determination".

This claim should have said *proposed* new phyla, to avoid the implication that they have been accepted as official taxonomy.

On p6, third column, Zayed2022 claims that vOTUs of "'Taraviricota' ... were, on average, the most abundant across most temperate and tropical waters", and of "'Arctiviricota' were, on average, the most abundant across most of the Atlantic Arctic waters (Fig. 4)."

Figures in the main paper show large histogram bars (Fig. 1B), large subtrees (Fig. 3A), and large pie chart wedges (Fig. 4) corresponding to the claimed new phyla.

No caveats are mentioned with regards to their use of the term "abundant". The overall presentation inevitably gives a reader the impression that the classification of these viruses as new phyla is unambiguous, and that they are highly abundant by any reasonable measure.

Missing vOTU abundance table

Given the main claims of the paper, the fundamental summary data is vOTU abundances per sample, with classifications of vOTUs. No such summary data is provided in the supplementary data.

Dominance is not defined

The word "dominance" (or a synonym) does not appear again in the main text or supplementary materials, leaving the basis of the main claim of the paper unspecified.

Abundance is not defined

No description of their abundance measure is given in the main text. The supplementary Methods text says that abundances were calculated by CoverM, (https://github.com/wwood/CoverM), but the details are not completely specified. It is described as follows: "For the vertical coverage

(i.e., for abundance estimation), reads that mapped at ≥90% ID over ≥75% of the read length were extracted using CoverM v0.2.0-alpha6, calculating the trimmed mean (tmean) for each contig... Only adjusted abundances of the ≥1-kb contigs were kept, and final abundances of the vOTUs were calculated by summing the adjusted abundances of the ≥1-kb contigs belonging to these vOTUs" (their Material and Methods under "Calculation of vOTU relative abundances"). It is not described how to calculate abundance of a "metataxon" or phylum from vOTU abundances, noting that quite different options could be reasonable, including the sum or mean per taxon. If the sum is used, a phylum with many species will tend to be more abundant. If the mean or maximum is used, a phylum with few species accounting for a small minority of reads may be more abundant than a phylum with many species and many more total reads. If the latter choice is made, this should be clearly explained, and a caveat should be stated in the claims.

Missing code for abundance calculation

No code is provided for calculating abundances from bowtie2 SAM files.

Missing CoverM reports

The reports generated by CoverM are not included in the supplementary data.

Reference

Zayed, A.A., Wainaina, J.M., Dominguez-Huerta, G., Pelletier, E., Guo, J., Mohssen, M., Tian, F., Pratama, A.A., Bolduc, B., Zablocki, O. and Cronin, D., 2022. Cryptic and abundant marine viruses at the evolutionary origins of Earth's RNA virome. *Science*, *376*(6589), pp.156-162.
