# Supplementary material for: Known phyla dominate the Tara Oceans RNA virome
Source: Virus Evol. 2023 Nov 8;9(2):vead063. doi: 10.1093/ve/vead063 (PMC10649353; doi:10.1093/ve/vead063)
Supplement: vead063_Supp [file vead063_supp.zip › Supplementary_Note_N2_RdRp_sequence_analysis_challenges.pdf]

Known phyla dominate the Tara Oceans RNA virome

Robert C. Edgar

Supplementary Note N2: Viral RdRp sequence analysis challenges

Viral RdRp protein

Viral RNA dependent RNA polymerase (RdRp) is the protein responsible for copying the genome of an RNA virus. Almost all RNA viruses make an RdRp protein (Wikipedia article on RdRp). Exceptions include RNA retroviruses, where the polymerase protein is a reverse transcriptase, and satellite viruses such as Hepatitis Delta Virus which require RdRp from another virus (the so-called helper virus) to be present to support replication. See Fig. SN2.1 for sketch of RdRp gene layout.

RdRp proteins are not named RdRp

Typically, the protein with RdRp function is not officially called RdRp. For example, in nidoviruses the RdRp protein is called Nsp9 or Nsp12 (Kirchdoerfer et al. 2019), as shown in Fig. SN2.2, and in the lambda 3 phage it is simply called the lambda protein (see PDB 1MUK). This complicates database searches by gene name.

Where does RdRp sequence terminate?

Sometimes, RdRp has a simple coding sequence (CDS) beginning with a START codon and ending at a STOP codon. In such cases, it is straightforward to identify RdRp as the nucleotide or translated amino acid sequence of this CDS (assuming of course that you know the genetic code of the host). However, things are not always that simple. RdRp often occurs in a longer ORF which codes for multiple proteins; for example in SARS-CoV-2 RdRp function is found in Nsp12 (Kirchdoerfer et al. 2019), one of several non-structural proteins coded in the long ORF1ab (21,555nt) which accounts for most of the genome (29,903nt). ORF1ab is translated into a long peptide (pp1ab), which is then split into mature proteins by a cleavage enzyme.

Multi-segment RdRp

In at least one family (narnaviruses), RdRp is not a single protein; it is assembled as a complex from preptides coded in multiple genome segments Sato et al. 2021).

Cleavage sites are hard to find

Unlike START and STOP codons, cleavage sites are not easily recognized by sequence analysis, and in fact are not known in many fully-sequenced genomes (Ferriol et al. 2016. Therefore, the beginning and end of the RdRp sequence in a genome or metagenomic contig may be difficult to

identify. In practice, this problem means that predicted RdRp sequences are often truncated or trimmed to shorter subsequences such as a partial domain.

RdRp domains

The RdRp protein is usually constructed from two or more domains. The term domain generally means a segment which folds independently and may be found in combination with other domains in different proteins, but the definition is not precise and may be used in different ways in different contexts.

Domain boundaries are fuzzy

It can be clear that some residues are in a particular domain (for example, the [G/S]DD motif is the palm domain). However, there are no sharp boundaries where two adjacent residues are definitively in different domains. Thus, in contrast to CDS which has clear boundaries (start / stop codons), domain boundaries are fuzzy.

Palm domain

The palm domain is found in all known viral RdRps (Jia and Gong 2019, te Velthuis 2014). Other named domains are usually present in an RdRp protein; for example, nidoviruses such as SARS-CoV-2 have a NiRAN domain at the N terminal before the palm, and reoviruses have an unnamed N-terminal domain before the palm and a bracelet domain after the palm. Regions outside of the palm domain are generally not well understood, and it is often not known whether they are essential for RdRp function or perform some other function.

Varying domains in RdRp

As the examples of nidoviruses (NiRAN+palm) and reoviruses (N-terminal+palm+bracelet) illustrate, the domain content of RdRp varies in different families, and except for the palm domain are often not well characterised. This adds to the difficulty of identifying the boundaries of the RdRp coding sequence in genomes which are far diverged from well-characterized viruses, and similarly in metagenomic contigs.

Permuted domains

Several families have permuted RdRp genes (Sabanadzovic2009, Ambrose2009, Ferrero2021, Gorbalenya2002) where the palm domain is permuted.

Palmprint

The palmprint is the segment which is long enough to contain all three catalytic motifs A, B and C, and no other sequence (Babaian and Edgar, 2022), see Fig. SN2.4. The sequence between A and B is called V1, and the sequence between B and C is called V2. In typical (unpermuted) palm domains, the motifs appear in ABC order and the palmprint thus extends from the start of motif A through the end of motif C, i.e. is A+V1+B+V2+C. In permuted palm domains, the motifs appear in CAB order and the palmprint then extends from the start of motif C to the end of motif B, i.e C+V3+A+V1+B.

Palmcore

The palmcore is the segment obtained by expanding the palmprint by exactly 150aa on each flank. This design balances the conflicting goals (a) include as much of the palm domain as possible, and (b) don't overflow into neighboring domains or genes.

Motif positions and lengths

Positions of the catalytic motifs are defined by the location of the one essential residue in each motif, i.e. ASP in motif A, GLY in motif B and ASP in motif C. The ASP in motif C is the first D in the canonical [G/S]D[D/N] motif. These definitions are somewhat arbitrary, but enable unambiguous identification of the palmprint and palmcore boundaries if the catalytic residues ASP-GLY-ASP can be located.

Motif A has two additional residues before the essential ASP and thus starts at ASP − 3.

Motif B has one additional residue before the essential GLY and thus starts at GLY − 1.

Motif C has two additional residues before the essential ASP and thus starts at ASP − 3.
Motif lengths

A has length 12, B has length 14 and C has length 8. As with the start positions, the lengths are somewhat arbitrary. These lengths were chosen based on experience in observing how well the sequence is conserved in diverged RdRps.

References

Ambrose, R. L. et al., Drosophila A virus is an unusual RNA virus with a T=3 icosahedral core and permuted RNA-dependent RNA polymerase, Journal of General Virology 2019 90(9) 2191-2200.

Babaian, A. and Edgar, R., 2022. Ribovirus classification by a polymerase barcode sequence. PeerJ, 10, p.e14055.

Ferrero, D.S.; Falqui, M.; Verdaguer, N. Snapshots of a Non-Canonical RdRP in Action. *Viruses* **2021**, *13*, 1260. https://doi.org/10.3390/v13071260.

Ferriol I, Silva Junior DM, Nigg JC, Zamora-Macorra EJ, Falk BW. Identification of the cleavage sites of the RNA2-encoded polyproteins for two members of the genus Torradovirus by N-terminal sequencing of the virion capsid proteins. Virology. 2016 Nov;498:109-115. doi: 10.1016/j.virol.2016.08.014. Epub 2016 Aug 25. PMID: 27567259.

Gorbalenya, A.E. et al. The Palm Subdomain-based Active Site is Internally Permuted in Viral RNA-dependent RNA Polymerases of an Ancient Lineage, Journal of Molecular Biology, 2002, 324(1), 47-62.

Jia, H. and Gong, P., 2019. A structure-function diversity survey of the RNA-dependent RNA polymerases from the positive-strand RNA viruses. *Frontiers in Microbiology*, *10*, p.1945.

Kirchdoerfer, R.N., Ward, A.B. Structure of the SARS-CoV nsp12 polymerase bound to nsp7 and nsp8 co-factors. *Nat Commun* **10**, 2342 (2019). https://doi.org/10.1038/s41467-019-10280-3.

Sead Sabanadzovic, Nina Abou Ghanem-Sabanadzovic, Alexander E. Gorbalenya,
Permutation of the active site of putative RNA-dependent RNA polymerase in a newly identified species of plant alpha-like virus, Virology, Volume 394, Issue 1, 2009.

Sato Y, Shahi S, Telengech P, Hisano S, Cornejo C, Rigling D, Kondo H, Suzuki N. A new tetra-segmented splipalmivirus with divided RdRP domains from Cryphonectria naterciae, a fungus found on chestnut and cork oak trees in Europe. Virus Res. 2022 Jan 2;307:198606. doi: 10.1016/j.virusres.2021.198606. Epub 2021 Oct 22. Erratum in: Virus Res. 2023 Jan 15;324:199013. PMID: 34688782.

te Velthuis AJ. Common and unique features of viral RNA-dependent polymerases. Cell Mol Life Sci. 2014 Nov;71(22):4403-20. doi: 10.1007/s00018-014-1695-z. Epub 2014 Aug 1. PMID: 25080879; PMCID: PMC4207942.

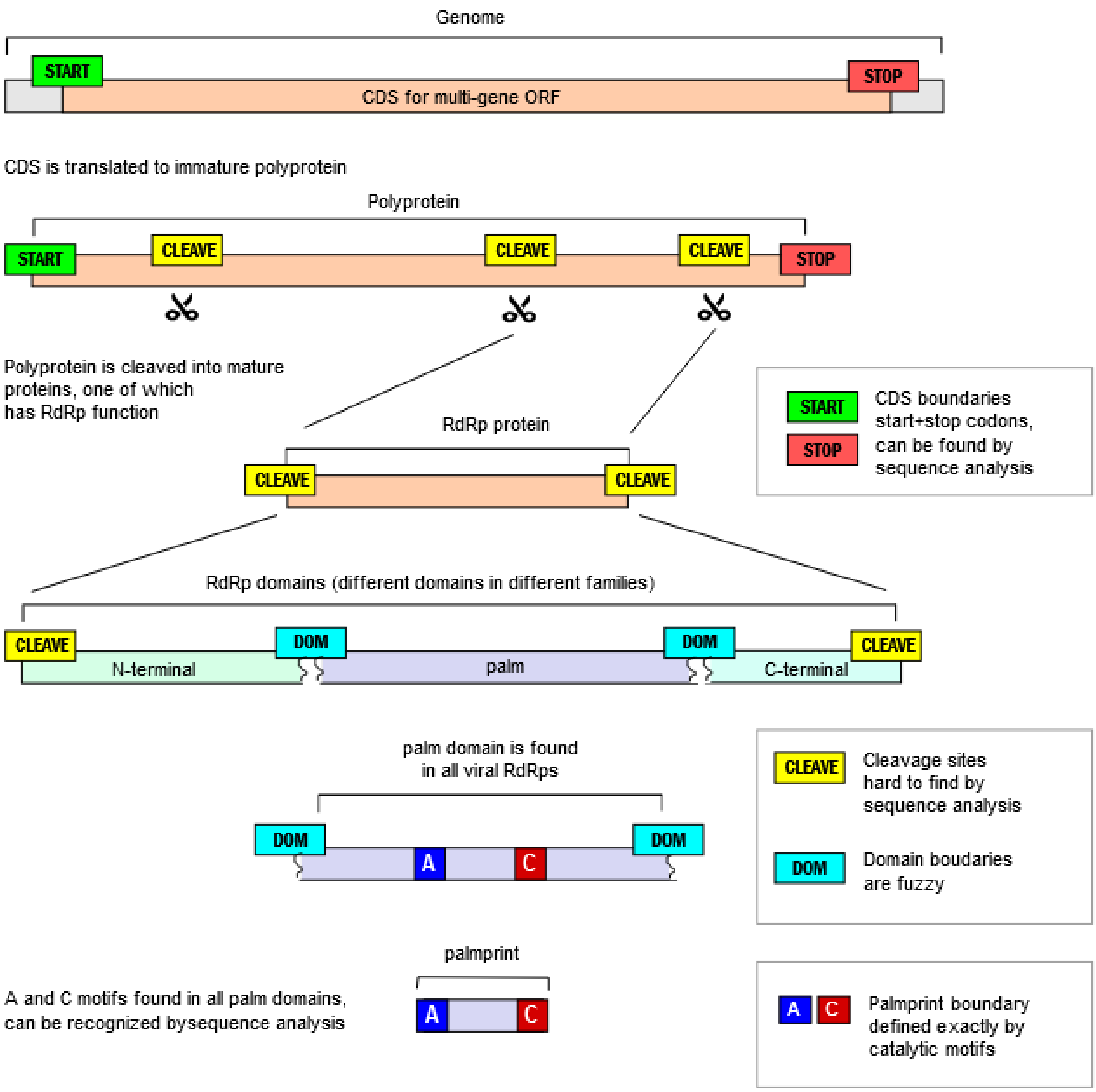

Fig. SN2.1. Typical layout of the RdRp gene.

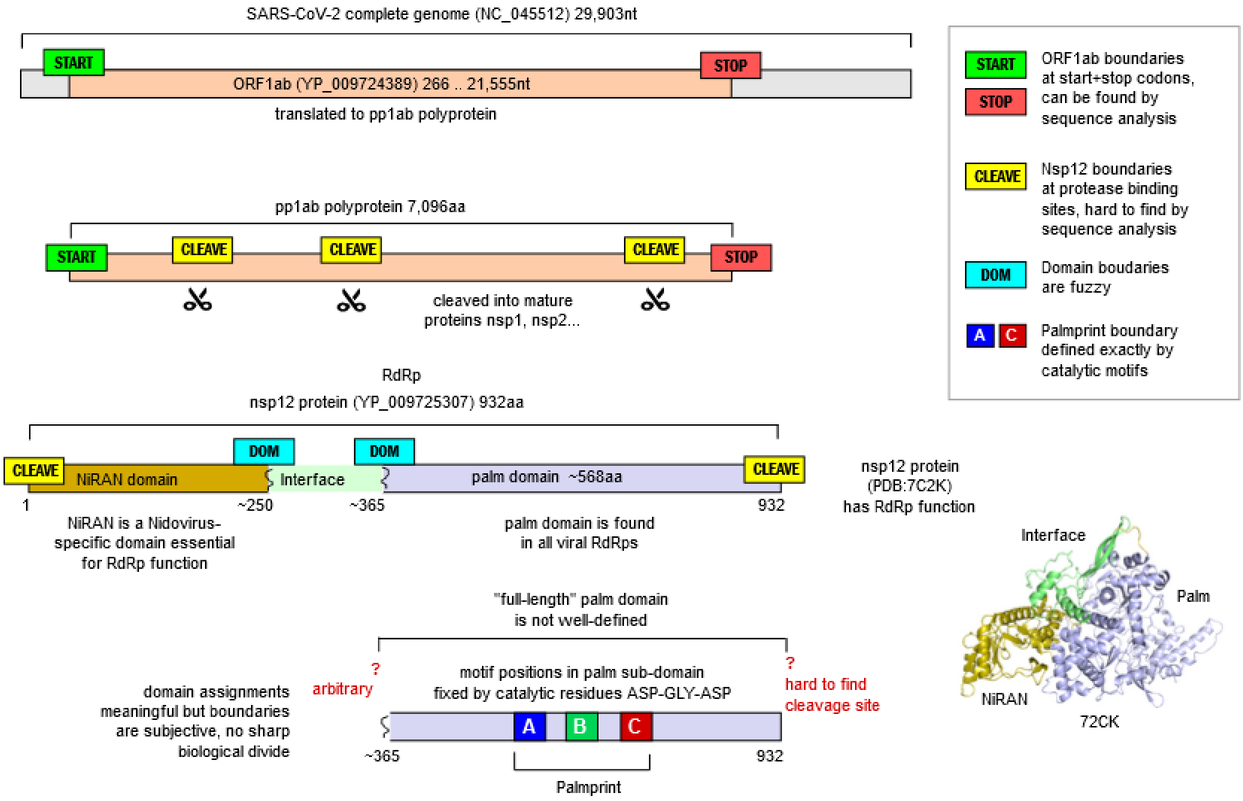

Fig. SN2.1. Layout of SARS-CoV-2 RdRp.

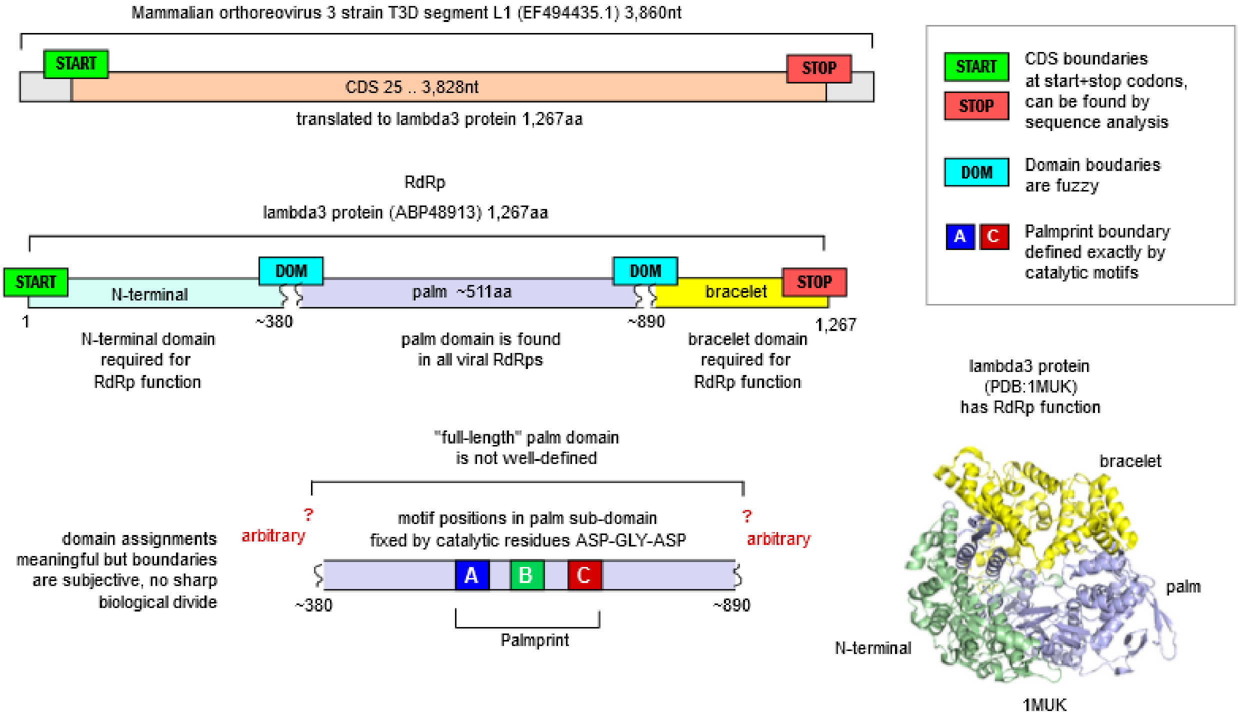

Fig. SN2.3. Layout of reovirus RdRp.

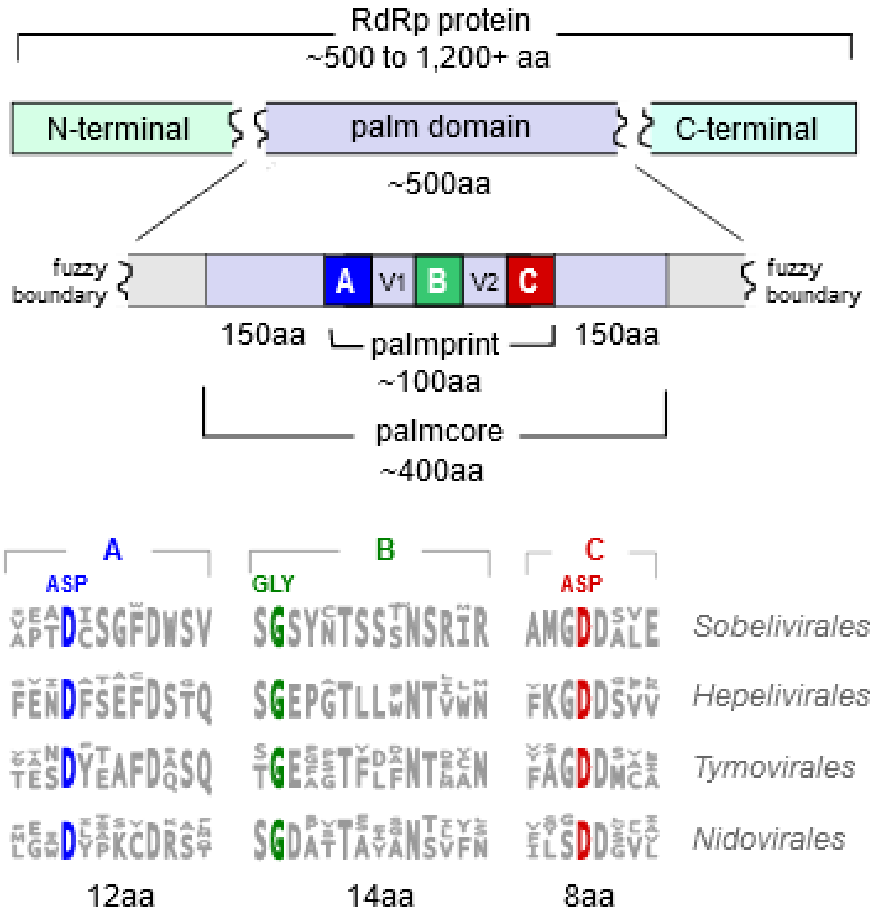

Fig. SN2.4. Defining the palmprint and palmcore by motifs A, B and C.
