# Supplementary material for: Known phyla dominate the Tara Oceans RNA virome
Source: Virus Evol. 2023 Nov 8;9(2):vead063. doi: 10.1093/ve/vead063 (PMC10649353; doi:10.1093/ve/vead063)
Supplement: vead063_Supp [file vead063_supp.zip › Supplementary_Note_N3_Distinguishing_viral_RdRp_from_homologs.pdf]

Known phyla dominate the Tara Oceans RNA virome

Robert C. Edgar

Supplementary Note N3: Distinguishing viral RdRp from homologs

Tara's protocol for detecting RdRp

In their Materials and Methods under 'Evaluation of authenticity and completeness of putative virus RdRps', Tara describe their protocol as follows:

> "Hits longer than 100 amino acids with a best match to an RdRp HMM and with a bitscore >=30 were kept as true positives for proteins containing the virus RdRp domain.
>
> Lower-scoring hits were manually inspected for presence of the seven canonical RdRp domain motifs...
>
> The domain sequences were manually inspected to ensure that predicted novel RNA virus phyla and classes derived from divergent RdRps are not false positives. Specifically, the seven canonical motifs (A–G, though motif E is missing in some bona fide RNA viruses) Te Velthuis et al. [sic], 2014) of virus RdRps were screened by searching for conserved regions in the consensus sequence of global alignments, and motif identity was confirmed based on HHPred homology searches and available literature."

While this procedure is reasonable for identifying putative viral RdRps, it is not definitive, certainly not to the standard that in my opinion should be required for a claim of novel phyla. The conservative assumption is that there will be residual, undetected false positives due to the following considerations.

HMM top hit

"Best match to an RdRp HMM" presumably means that the search was performed with HMMs for RdRps and also close homologs such as viral reverse transcriptases and Group II introns. As such, it is a top-hit / nearest-neighbor test which may give false-positive assignments because top hits are not necessarily evolutionary neighbors (Koski and Golding, 2001), and RdRp HMMs can have low E-values to non-viral homologs. For example, WP_011745321_1 Group II intron of *Chlorobium phaeobacteroides* has E=6.5e-07 to PFAM PF00680 RdRP_1.

E-values are more robust than bit scores

Tara's use of a bit score threshold is unconventional, why were E-values not used? A bit score of 30 is low and typically corresponds to a high E-value; the corresponding E-value should therefore have been quoted and the choice of bit score threshold should have been explained. Presumably, some or all of the claimed novel phyla are among the most diverged and hence lowest-scoring hits, but supporting data is not provided so this cannot be verified.

Identification of canonical motifs

Manual inspection of sequences cannot reliably identify all seven catalytic motifs in highly diverged RdRp sequences because motifs D, E, F and G are well-conserved in structure but diverge rapidly in sequence. Even within a known order, in my experience it is often difficult or impossible to identify D, E, F and G motifs without structure (see example in Fig. SN3.1). Given the very low sequence conservation of these motifs, I doubt that HHPred can accurately align them, and even if can be accuracy aligned in cases where they are present, what in the alignment would distinguish a structural motif from a distinctly different secondary structure at the same position in the amino acid sequence? Tara should have deposited the relevant alignments and described the criteria used to distinguish present from absent motifs to enable independent review of their conclusions.

Most of the predicted structures deposited by Tara have missing or malformed motifs (Supp Note. N6).

I could not find a statement in te Veltuis 2014 saying that motif E is absent in any viral RdRp. Regardless, I believe that motif E is definitively present in 3mmp:G (see Supplementary Note N6, especially Fig. SN6.4).

These observations call into question the robustness of Tara's protocol for identifying motifs. Tara should have provided a list of hits they considered to be low-scoring, together with their inferred motifs and functional assignments, to enable independent review.

Motifs cannot definitively distinguish RdRp from cellular homologs

While rules of thumb can be applied to motif sequences, e.g. GLY-ASP-ASP (GDD) in motif C is characteristic of RdRp while ALA-ASP-ASP (ADD) is characteristic of reverse transcriptases, there is to the best of my knowledge no reliable method for distinguishing viral RdRp from a cellular homolog given its sequence and structure alone because all canonical motifs are present in both RdRp and Group II introns (Fig. SN3.2), and there are exceptions to sequence-based rules of thumb. A rule of thumb is anecdotal evidence, and as such it is not definitive -- seeing many white swans does not prove that all swans are necessarily white, or that a black bird cannot be a swan. Similarly, rules based on a limited sample of known RdRps and homologs are not definitive for assigning function to an RdRp-like sequence which is sufficiently diverged from anything known to justify assignment to a putative new phylum. GDD=RdRp / ADD=RT for motif C is probably the best-known and most reliable rule, but there are counter-examples. AOY33888 (RdRp of squash vein yellowing virus) has ALA-ASP-ASP in its C motif, and conversely WP 014123481 (group II intron of bacterium *Tetragenococcus halophilus*) has GLY-ASP-ASP (Fig. SN3.3).

References

Koski, L. B. and Golding, G. B. (2001). The closest blast hit is often not the nearest neighbor. Journal of molecular evolution, 52(6):540–542.

te Velthuis, A. J. (2014). Common and unique features of viral rna-dependent polymerases. Cellular and molecular life sciences, 71(22):4403–4420.

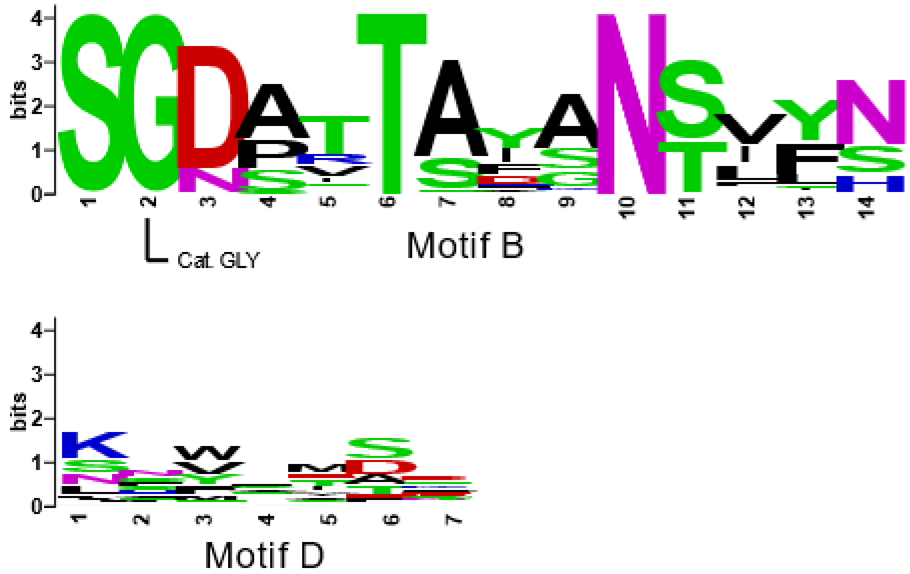

Fig. SN3.1. Sequence conservation of motifs B and D in *Nidovirales*.
Sequence logos by weblogo (Crooks et al. 2004) from a Muscle5 alignment. As seen here, even within a single order motif D is so weakly conserved as to be effectively unrecognizable, while motif B is highly conserved in sequence and readily recognized visually or by a PSSM. Between different phyla, all motifs are challenging to recognize by sequence, as shown by the Wolf2018 misalignments of A, B and C reported in the Muscle5 paper (Edgar 2022). See Table SN3.1 for motif sequences.

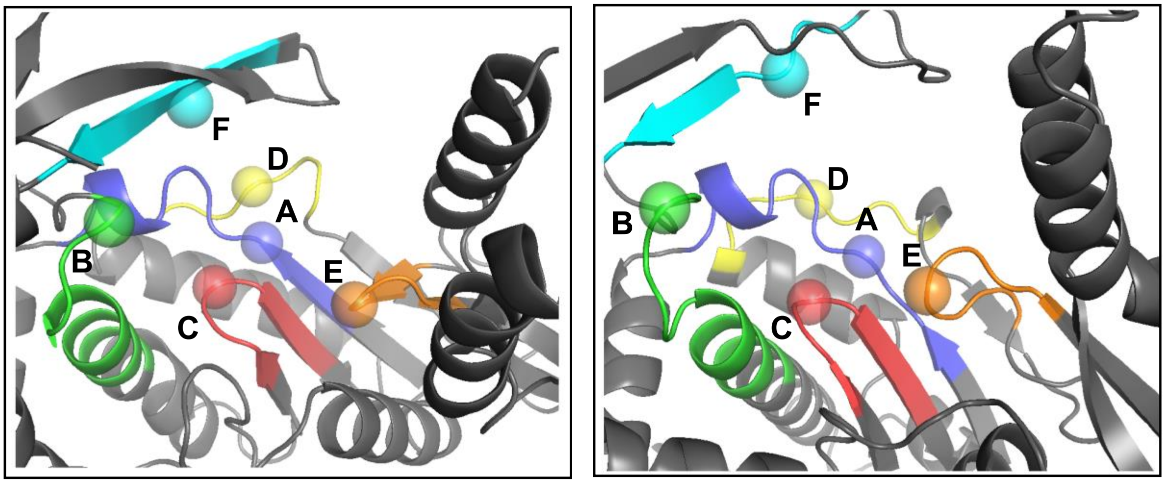

RdRp 7c2k

Group II intron 6ar3-A

Fig. SN3.2. Structural alignment of viral RdRp to a cellular homolog.
On the left is the palm domain of SARS-Cov-2 RdRp (PDB:7c2k) aligned to *Geobacillus stearothermophilus* group II intron (right, PDB:6ar3) showing that the essential conserved motifs A through F have similar conformations between RdRp and cellular homologs.

```
>AOY33888.1 RdRp [Squash vein yellowing virus]
   A:2553-2564(16.4)       B:2618-2631(21.1)       C:2656-2663(14.4)
   IDCDASRSDSSL      <77> SGQPPPVVDNTLLS    <52> CNADDLII  [111]
   .|.| || ||||           ||||  ||||||           |  ||| |
   CDADGSQFDSSL           SGQPSTVVDNTLMV         aNGDDLlI
Score 51.9, Patatavirales (Duplorna +19.1)


>WP_014123481.1 Group II intron [Tetragenococcus halophilus]
   A:141-152(16.5)       B:197-210(17.1)       C:226-233(10.7)
   VDCDLKSYFDTI    <68> QGSPLSPLLANVYL  <43> RYGDDFII  [93]
   |++||+|+||+|         ||||||| | |+|+       +| ||. +
   vsiDlekAfDrv         qGdPLSnilfslvl       aFADDivv
Score 44.3, RT (RT2 +0.0)
```

Fig. SN3.3. Misleading C motifs.

Palmscan alignments of RdRp AOY33888.1 and Group II intron WP_014123481.1 showing that AOY33888.1 has ALA-ASP-ASP (ADD) in its C motif, which is typically found in reverse transcriptases, and conversely that WP_014123481 has GLY-ASP-ASP (GDD) which is typically found in RdRps. These misleading motifs could be explained by sequence errors in GenBank, but sequence errors can also occur in metagenomic assemblies. Therefore, motifs can be informative but are not definitive for distinguishing RdRp from cellular homologs.

Virus	*Motif B*	*Motif D*
AB753015.2 Alphamesonivirus 3	SGNSRTADGNSLLH	SHYMIAQ
AF180391.2 Deltaarterivirus hemfev	SGDPVTSIANTIYS	KTVITSN
AF227196.2 Gill-associated virus	SGDGATAIKNSHCN	KIFVRDE
AF353511.1 Porcine epidemic diarrhea virus	SGDATTAYANSVFN	VFMSASK
AJ271965.2 Alphacoronavirus 1	SGDGTTAYANSAFN	NNVFMST
AY274119.3 Severe acute respiratory syndrome-related coronavirus	SGDATTAYANSVFN	NNVFMSE
AY427798.1 Bovine torovirus	SGDATTAHSNTFYN	KSWATTG
AY567487.2 Human coronavirus NL63	SGDASTAYANSIFN	VFMSTSK
AY585228.1 Betacoronavirus 1	SGDATTAFANSVFN	KCWVEHD
AY613950.1 Severe acute respiratory syndrome-related coronavirus	SGDATTAYANSVFN	NNVFMSE
DQ458789.2 Alphamesonivirus 1	SGNSRTADGNSLLH	SHYMIAQ
DQ648858.1 Scotophilus bat coronavirus 512	SGDATTAYANSVFN	NNVFMSA
DQ898157.1 White bream virus	SGDSTTAFSNSFYN	LGCYVDP
EU420138.1 Miniopterus bat coronavirus 1	SGDATTAYANSVFN	NNVFMST
EU420139.1 Miniopterus bat coronavirus HKU8	SGDATTAYANSVFN	NNVFMST
EU487200.1 Yellow head virus	SGDGATAIKNSHCN	KIFVRDE
FJ376619.2 Bulbul coronavirus HKU11	SGDATTAYANSVFN	KCWTETD
FJ376622.1 Munia coronavirus HKU13	SGDATTAYANSVFN	KCWTETD
GBBW01007738.1 Aplysia abyssovirus 1	SGNSRTKTVNGNIN	VEMKKEK
GU002364.2 Fathead minnow nidovirus 1	SGDSTTAFSNSFYN	LGCAVDV
JQ065043.2 Coronavirus HKU15	SGDATTAYANSVFN	KCWTETD
JQ065044.1 White-eye coronavirus HKU16	SGDATTAYANSVFN	KCWTETD
JQ065047.1 Night heron coronavirus HKU19	SGDATTAYANSVFN	KCWIETD
JQ065048.1 Wigeon coronavirus HKU20	SGDATTAYANSVFN	KCWVESD
JQ065049.1 Common moorhen coronavirus HKU21	SGDATTAYANSVFN	KCWTETD
JQ860350.1 Porcine torovirus	SGDATTAHSNTFYN	KSWSASG
JQ957872.1 Alphamesonivirus 5	SGNSRTADGNSLLH	SHYMMAQ
JQ957873.1 Alphamesonivirus 9	SGNSRTADGNSFLH	SKYAIAT

JQ957874.1 Alphamesonivirus 8	SGNSRTADGNSLLH	SYYMIAQ
JQ989270.1 Bat coronavirus HKU10	SGDASTAYANSVFN	NNVFMST
JX473849.1 Iotaarterivirus kibreg 1	SGDPVTSISNTIYS	LGFKTDP
KC787630.1 Zetaarterivirus ugarco 1	SGDPITSIANTIYS	KTVITTN
KC787658.1 Etaarterivirus ugarco 1	SGDPVTSTSNTIYS	KTVITSD
KC807171.1 Alphamesonivirus 2	SGNSRTADGNSLLH	SHYMISL
KF636752.1 Bat Hp-betacoronavirus Zhejiang2013	SGDATTAYANSVFN	VFMSEAK
KJ125489.1 Alphamesonivirus 4	SGNSRTADGNSLLH	SHYMIAQ
KJ473806.1 Myotis ricketti alphacoronavirus Sax-2011	SGDATTAYANSVFN	NNVFMST
KJ473807.1 Rhinolophus ferrumequinum alphacoronavirus HuB-2013	SGDATTAYANSVFN	VFMSTSK
KJ681496.1 Chinook salmon nidovirus 1	SGDSTTAFSNSIYN	KAWAASG
KM110938.1 Thetaarterivirus mikelba 1	SGDPVTSIANTVYS	LGFVVDR
KM589359.1 Bovine nidovirus 1	SGDATTAFSNSIYN	IFTQVDR
KP026921.1 Lambdaarterivirus afriporav	SGDPVTSICNTIYS	LGFVTDP
KP126831.1 Iotaarterivirus debrazmo	SGDPVTSVANTIYS	LGFKTDP
KR139839.1 Iotaarterivirus pejah	SGDPVTSIANTIYS	KTVVTTN
KR862307.1 Epsilonarterivirus safriver	SGDPCTSIANTIYS	LGFKVDP
KT966491.1 Alphamesonivirus 6	SGNSRTADGNSLLH	SKYSIAT
KT966495.2 Alphamesonivirus 7	SGNSRTADGNSFLH	SKYLIAN
KX184715.1 Shingleback nidovirus 1	SGDATTAFANTLYN	IHTTVDE
KX883628.1 Charybnivirus 1	SGNSITALNNSLAA	YFVSRAD
KX883629.1 Turrinivirus 1	SGNSKTAPGNSIMH	SNYHFTN
KX883637.1 Sectovirus 1	SGDATTAFSNTLYN	LHTTVDE
KX883638.1 Infratovirus 1	SGDATTAFGNTLYN	KAWQGDT
KY056254.1 Alphamesonivirus 10	SGNSRTADGNSLLH	SHYMIAQ
KY073745.1 NL63-related bat coronavirus strain BtKYNL63-9b	SGDATTAYANSVFN	NNVFMST
KY352407.1 Severe acute respiratory syndrome-related coronavirus	SGDATTAYANSVFN	NNVFMSE
KY369959.1 Alphamesonivirus 11	SGNSRTADGNSLLH	STYDIPN
KY369967.1 Betaarterivirus timiclar	SGDPITSISNTIYS	LGFKTDP
KY370046.1 Myodes coronavirus 2JL14	SGDATTAFANSVFN	NNVFMSE
KY967715.1 Suncus murinus coronavirus X74	SGDASTAYANSVFN	NGVFMSN

M95169.1 Avian coronavirus	SGDATTAYANSVFN	KCWVEPD
MF351889.1 Morelia tobanivirus 1	SGDATTAFANTLYN	KAWESKG
MF685025.1 Berisnavirus 1	SGDATTAFANTLYN	KAWESDG
MG600020.1 Halfbeak nidovirus 1	SGDACTCHTNTIFN	KSHSAKL
MG600023.1 Ptyasnivirus 1	SGDANTTLANTVQS	KTTISKP
MG923574.2 Alphacoronavirus BT020	SGDATTAYANSVFN	VFMSESK
MH447987.1 Sicregavirus nixi	SGDPFTSIVNSCYT	KSFVSKH
MK359255.1 Goose coronavirus CB17	SGDATTAYANSVFN	KCWTEPD
MK472067.1 Alphacoronavirus WA1087	SGDATTAYANSVFN	VFMSASK
MK611985.1 Alphapironavirus bona	SGDATTAYANSAFN	KCWTEAD
MK720944.1 Alphacoronavirus HKU33	SGDATTAYANSVFN	VFMSTSK
MK956105.1 Botrylloides leachii nidovirus	SGSSKTANGNSYSH	SNYVFPR
MN161561.1 Sertovirus cona	SGDATTAYANSIYN	IHAPVDR
MN161566.1 Septovirus foka	SGDATTAFANTLYN	KAWESEG
MN161572.1 Infratovirus latu	SGDATTAFANTLYN	KAWQGED
MN714662.1 Insemevirus tami	SGNPCTADGNSNIH	SNFNMTP
MN714663.1 Tofonivirus foami	SGNSRTADGNSGIH	YVIEPEK
MN961271.1 Tocinivirus aphisi	SGNSRTADGNSFCH	SNYDMPM
MT663548.1 Alphacoronavirus AMALF	SGDATTAYANSVFN	NNVFMST
MT907511.1 Nimanivirus lahi	SGDGATAIKNSHCN	KFFYTEV
MT997159.1 Vebetovirus paba	SGDATTAFANTLYN	KAWEADG
X53459.3 Alphaarterivirus equid	SGDPITSISNTIYS	LGFKVDP

Table. SN3.1. Motif B and D sequences for nidoviruses.

Obtained from Muscle5 alignment to manual annotation of PDB:7c2k.
