# Supplementary material for: Known phyla dominate the Tara Oceans RNA virome
Source: Virus Evol. 2023 Nov 8;9(2):vead063. doi: 10.1093/ve/vead063 (PMC10649353; doi:10.1093/ve/vead063)
Supplement: vead063_Supp [file vead063_supp.zip › Supplementary_Note_N4_Megataxonomy_and_incorrect_97pct_accuracy_claim.pdf]

Known phyla dominate the Tara Oceans RNA virome

Robert C. Edgar

Supplementary Note N4: Tara's "megataxonomy" and incorrect claim of 97% accuracy

Megacluster workflow

Tara's "megataxonomy" classification method is sketched in Fig. SN4.1. Translated RdRp sequences from Tara contigs were combined with RdRps from Yangshan Deep-Water Harbour and GenBank. The combined RdRps were reduced to 13,109 non-redundant centroid sequences at 50% identity by UCLUST (Edgar, 2010). The centroids were then clustered by MCL (Enright et al., 2002) from a matrix of pair-wise BLASTP bit scores, giving 19 "megaclusters". Each megacluster was assigned a "megataxon" based on the majority taxon or taxa according to annotations of its GenBank sequences.

Incorrect claim of 97% agreement with ICTV taxonomy

Tara claimed that their MCL clusters "nearly completely recapitulated the previously established phylogeny-based ICTV-accepted taxonomy at the phylum and class ranks (97% agreement)". This claim is apparently based on the ARI=0.97 value reported in their Fig. 1B. However, the agreement underlying this claim is not the fraction of correct classifications of phylum and class with GenBank taxonomy annotations as implied by the statement; rather, it is the adjusted Rand index obtained by comparing "megataxa" and megaclusters. "Megataxa" do not correspond to phylum and class separately or to phylum and class together; rather they are a heterogeneous collection of phyla, sub-phyla, polyphyletic groups (e.g. "Lenarviricota, others"), classes, unassigned ranks (e.g. "Wei-like"), and sub-families. With three exceptions (*Chrymotiviricetes*, *Vidaverviricetes* and *Allassoviricetes*), "megataxa" cannot be used to classify to ICTV class rank.

Classifier over-fitting and generalising to new data

Tara's *ad hoc* MCL clustering classifier was tuned to all available training data, which surely results in extreme over-fitting because, as with the 3D structure network (Supp. Note N5), there are no stated prior restraints on variations which can be tried, including the choice of distance metric, choice of clustering method, choice of clustering parameters (e.g. inflation value), and choice of ICTV taxa assigned to a cluster. The number of possible variations is thus astronomical, and the opportunities for over-fitting are therefore for all practical purpose unlimited. Given that a high degree of over-fitting should be assumed, it follows that the agreement between megaclusters and "megataxa" is not predictive of agreement when classifying new, unlabelled sequences (Santos et al., 2018), even if it is accepted that "megataxa" defined by this approach should supplant ICTV taxa.

Monophyletic taxa

In taxonomy generally, and per ICTV rule 3.3.1 specifically, monophyly is a fundamental requirement for defining taxa. However, it is textbook knowledge that clustering cannot reliably infer phylogenetic trees or monophyletic groups, with one exception based on unrealistic assumptions (the correct tree can be reconstructed by UPGMA if the molecular clock is ultrametric and true evolutionary distances can be calculated). This result was established in the 1960s in the first literature to consider mathematical and algorithmic applications to phylogenetics, and has been universally accepted since then. See Chapter 10 in "Inferring Phylogenies" (Felsenstein, 2004) for history, methodological survey, and references. MCL is a generic clustering method, and therefore cannot reliably predict monophyletic groups. Adopting a MCL-based approach to classification would therefore require abandoning monophyly as a standard, without providing alternative classification principles.

Lack of classification principles

If a future study finds that MCL with inflation 1.1 based on BLASTP bit scores produces quite different clusters from Tara's when new data is added, which seems inevitable, then how to proceed? Should Tara's "megataxa" be abandoned? Should a different clustering method or similarity score be tried? Tara offers no guiding principles for answering such questions.

References

Edgar, R.C., 2010. Search and clustering orders of magnitude faster than BLAST. *Bioinformatics*, *26*(19), 2460-2461.

Enright, A. J., Van Dongen, S., and Ouzounis, C. A. (2002). An efficient algorithm for large-scale detection of protein families. Nucleic acids research, 30(7):1575-1584.

Felsenstein, J. (2004). Inferring Phylogenies. Sinauer Associates, Sunderland, MA.

Santos, M. S., Soares, J. P., Abreu, P. H., Araujo, H., and Santos, J. (2018). Cross-validation for imbalanced datasets: avoiding overoptimistic and overfitting approaches [research frontier]. IEEE Computational Intelligence Magazine, 13(4):59-76.

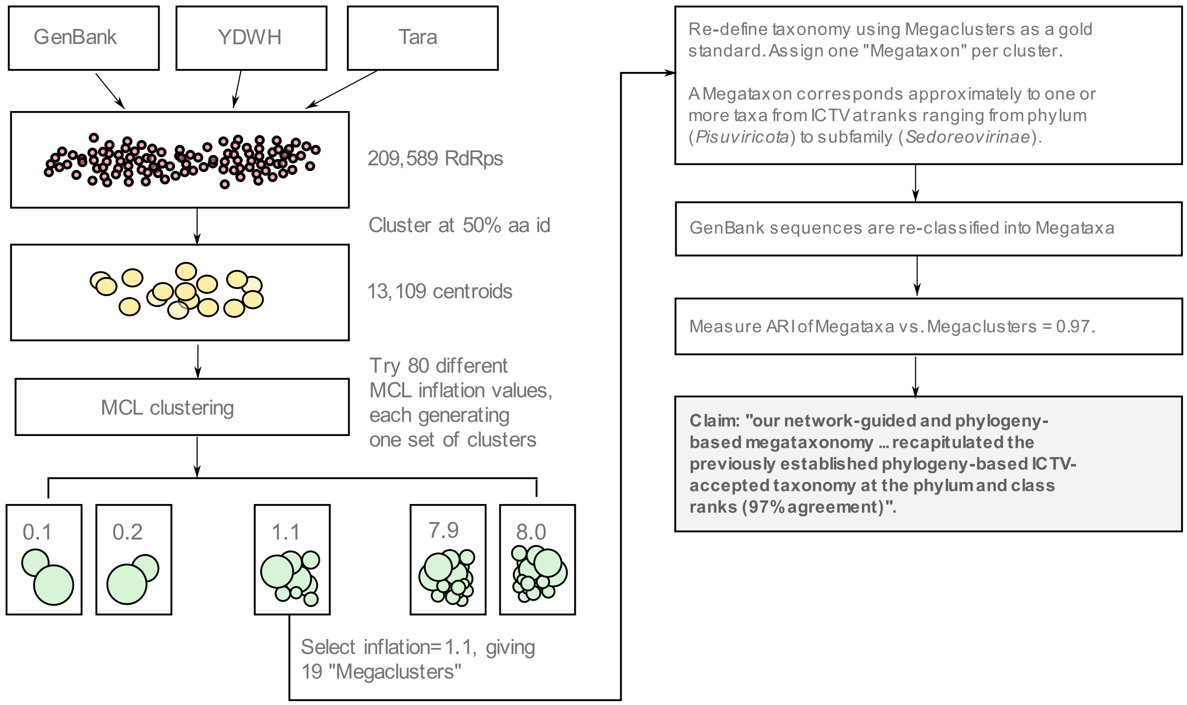

Fig. SN4.1. Tara's "megacluster" workflow and incorrect claim of 97% agreement with ICTV taxonomy.
