# Supplementary material for: Known phyla dominate the Tara Oceans RNA virome
Source: Virus Evol. 2023 Nov 8;9(2):vead063. doi: 10.1093/ve/vead063 (PMC10649353; doi:10.1093/ve/vead063)
Supplement: vead063_Supp [file vead063_supp.zip › Supplementary_Note_N5_Structure_network_analysis.pdf]

Known phyla dominate the Tara Oceans RNA virome

Robert C. Edgar

Supplementary Note N5: Tara's structure network analysis

Structure network method

Tara's Fig. 3B shows a "structure network" which, according to the figure caption, is informative for "...inferring the early history of orthornavirans". As described in their Material and Methods under "3D structure network analysis", pair-wise structural alignments were constructed using Matras v1.2 (Kawabata, 2003). For each pair, the superfamily reliability score was used as a distance. A network visualisation was generated by cytoscape (Shannon et al., 2003) from these distances, using the using the "Edge-weighted Spring" layout.

Claim of informative for phylogenetic inference unsupported

No citations to previous applications of networks to phylogenetics or taxonomy are given, no justification is given from theoretical considerations, and no validation is reported which assesses the accuracy of inferences made from this type of network. The remarkable claim that this *ad hoc* method is informative for deep phylogenetic inference is thus not supported by any evidence.

Inference methods not described

The procedures and criteria used to make inferences from the network are not explained; conclusions are simply presented as *faits accomplis*.

Lack of rational principles

Why Matras scores and cytoscape? Could DALI (Holm and Sander, 1995) Z-scores or TM-align (Zhang and Skolnick, 2005) TM scores be used instead? Could the "Compound Spring" or "Prefuse Force" layouts be used, or is "Edge-weighted Spring" superior for some reason? Could the network be generated using Gephi (gephi.org) or Wandora (wandora.org)? More generally, which combinations of similarity scores and network construction methods are valid, and which are invalid? What objective criteria enable phylogenetic inferences from these networks? What types of hypotheses can be robustly confirmed or contradicted? How can the accuracy of these conclusions be assessed? In the absence of a framework for delivering evidence-based answers to questions like these, this type of method cannot support a rational approach to classification.

References

Holm, L. and Sander, C. (1995). Dali: a network tool for protein structure comparison. Trends in biochemical sciences, 20(11):478–480.

Kawabata, T. (2003). Matras: a program for protein 3d structure comparison. Nucleic acids research, 31(13):3367–3369.

Shannon, P., Markiel, A., Ozier, O., Baliga, N. S., Wang, J. T., Ramage, D., Amin, N., Schwikowski, 12
B., and Ideker, T. (2003). Cytoscape: a software environment for integrated models of biomolecular interaction networks. Genome research, 13(11):2498–2504.

Zhang, Y. and Skolnick, J. (2005). TM-align: a protein structure alignment algorithm based on the TM-score. Nucleic acids research, 33(7):2302–2309.
