# Supplementary material for: Known phyla dominate the Tara Oceans RNA virome
Source: Virus Evol. 2023 Nov 8;9(2):vead063. doi: 10.1093/ve/vead063 (PMC10649353; doi:10.1093/ve/vead063)
Supplement: vead063_Supp [file vead063_supp.zip › Supplementary_Note_N6_Predicted_RdRp_structures_and_motifs.pdf]

Known phyla dominate the Tara Oceans RNA virome

Robert C. Edgar

Supplementary Note N6: Predicted structures and motifs for claimed new phyla

RdRp structure

The viral RdRp protein typically has two or more domains. The domain content varies in more distantly related viruses, with only the palm domain universally present (Supp. Note N2).

RdRp palm domain structure and conserved motifs

The viral RdRp palm domain belongs to the palm domain superfamily, with close homologs in several families of cellular palm domain proteins including group II introns. There are six essential motifs in the catalytic core of the polymerase palm domain (Lang et al., 2012), conventionally denoted by letters A through F which usually appear in order FABCDE in the primary sequence, though permuted variants are also known (e.g. (Gorbalenya et al., 2002)). A seventh motif G is sometimes included; I do not consider it further here because its structural conservation is less clear. Motifs F, A, B and C each have one conserved catalytic residue (F=ARG, A=ASP, B=GLY and C=ASP). Within a phylum, the A, B and C motifs are sufficiently well conserved to be recognizable by sequence (Babaian and Edgar, 2022), while the remaining motifs are much more variable in primary sequence (Fig. SN6.6). Between different phyla, motifs are generally not recognizable by primary sequence similarity with the exception of GLY-ASP-ASP in motif C which has a few common variants such as SER-ASP-ASP in SARS-Cov-2. All six motifs are found in all solved structures for palm domain polymerases and reverse transcriptases (see (Lang et al., 2012) for motif coordinates in structures known in 2012). They are well conserved in structure, aligning well between viral RdRp and non-viral homologs including group II introns, as shown in Fig. SN6.1.

RdRp cannot be distinguished from cellular homologs by its sequence and structure alone

While rules of thumb can be applied to motif sequences, e.g. GLY-ASP-ASP in motif C is characteristic of RdRp while ALA-ASP-ASP is characteristic of reverse transcriptases, there is to the best of my knowledge no reliable method for distinguishing viral RdRp from a cellular homolog given its sequence and structure alone because there are exceptions. For example, AOY33888 (RdRp of squash vein yellowing virus) has ALA-ASP-ASP in its C motif, and conversely WP 014123481 (group II intron of bacterium *Tetragenococcus halophilus*) has GLY-ASP-ASP. If six new virus phyla can be discovered in Tara's data, then surely one or more new families of group II introns, or some other close cellular homolog, could also be discovered. Given that the diversity of palm domain proteins is known only sparsely at the present time, it is

simply not possible to distinguish with certainty a highly diverged RdRp from, say, a highly diverged group II intron by sequence and structure alone.

Predicted RdRp structures for Tara's claimed novel phyla

According to Tara's Material and Methods under 3D structure network analysis, they "predicted the 3D structures for the new megataxa from their representative primary amino acid sequences (the longest sequence with no ambiguous residues (i.e., no 'X's in the primary sequence) per megatxon) using Phyre2 in the 'Normal' mode". They deposited five structures, one for each "phylum" in cyverse: https://de.cyverse.org/anon-files//iplant/home/shared/iVirus /ZayedWainainaDominguez-Huerta RNAevolution Dec2021/Predicted 3D Structures/. I downloaded the pdb files for these structures and aligned them to SARS-CoV-2 RdRp (PDB:7c2k) using pymol (https://www.pymol.org) (see Supp Note. N7 for tutorial on aligning and evaluating RdRp structures). The predicted structure for "Taraviricota" aligned well and in my judgment appears consistent with a polymerase or reverse transcriptase in the palm domain superfamily. However, structures for the other four claimed phyla are obviously truncated and malformed. Fig. SN6.2 summarizes their alignments to CoV. These four predicted structures range in length from 142 aa ("Arctiviricota") to 211 aa ("Wamoviricota") and are thus much shorter than a complete RdRp domain which ranges from a minimum of more than 500 aa to a maximum of >1,200 aa; in fact, they are too short to span even a complete palm domain (Supp. Note N2). The catalytic cores of the predicted palm domains are truncated such that one or more essential motifs are missing, most obviously in "Pomiviricota" which lacks motifs A, B and C and F. In "Wamoviricota" and "Parexenoviricota" roughly half the catalytic core is present but obviously malformed, as shown in Fig. 11 in the main text. For example, in "Parexenoviricota", one strand of the anti-parallel beta sheet of motif C is replaced by one helix turn and in "Wamoviricota", the alpha helix of motif B, which has five or more complete turns in all known structures, has a single turn followed by a loop. Therefore, if these predicted structures are substantially correct, they are sufficiently different from known palm domain polymerases to contradict the hypothesis that they are viral RdRp. Conversely, if the predictions have substantial errors and the true structures closely resemble known viral RdRps, then the predicted structures are sufficiently defective to undermine inferences of function and phylogenetic relationships by comparison with solved palm domain structures.

Annotation of predicted structures in Tara's Fig. S5

My analysis of the predicted structures conflicts with Tara's Fig. S5, reproduced with added notes as my Fig. SN6.5. Tara marks 12 motifs as present which in fact are unambiguously absent due to truncation of the palm domain. Motif E is annotated as "naturally absent" in Lenarviricota PDB:3mmp-G, but in fact this essential motif is found at residue 397 as shown in my Fig. SN6.4.

References

Babaian, A. and Edgar, R., 2022. Ribovirus classification by a polymerase barcode sequence. PeerJ, 10, p.e14055.

Edgar, R.C., 2022. Muscle5: High-accuracy alignment ensembles enable unbiased assessments of sequence homology and phylogeny. Nature Communications, 13(1), p.6968.

Crooks GE, Hon G, Chandonia JM, Brenner SE WebLogo: A sequence logo generator, Genome Research, 14:1188-1190, (2004).

Gorbalenya, A. E., Pringle, F. M., Zeddam, J.-L., Luke, B. T., Cameron, C. E., Kalmakoff, J., Hanzlik, T. N., Gordon, K. H., and Ward, V. K. (2002). The palm subdomain-based active site is internally permuted in viral RNA-dependent RNA polymerases of an ancient lineage. Journal of molecular biology, 324(1):47–62.

Lang, D. M., Zemla, A. T., and Zhou, C. L. E. (2012). Highly similar structural frames link the template tunnel and NTP entry tunnel to the exterior surface in RNA-dependent RNA polymerases. Nucleic Acids Research, 41(3):1464–1482.

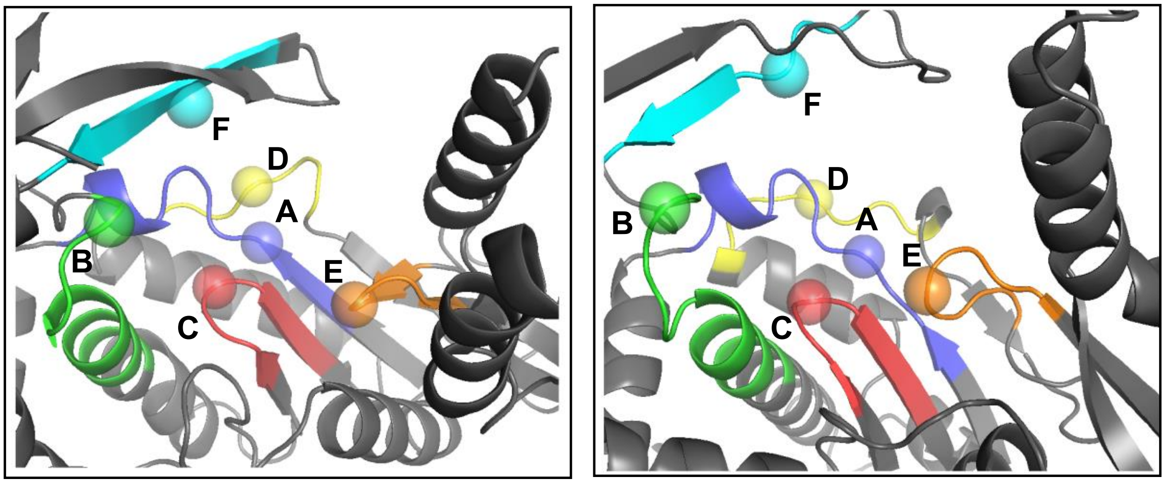

RdRp 7c2k

Group II intron 6ar3-A

Fig. SN6.1. Structural alignment of viral RdRp to a cellular homolog.
On the left is the palm domain of SARS-Cov-2 RdRp (PDB:72ck) aligned to *Geobacillus stearothermophilus* group II intron (right, PDB:6ar3) showing the essential conserved motifs A through F. Motif sequences and structural conformations are sometimes suggestive of assignment to viral RdRp or to a cellular homolog, but are never definitive.

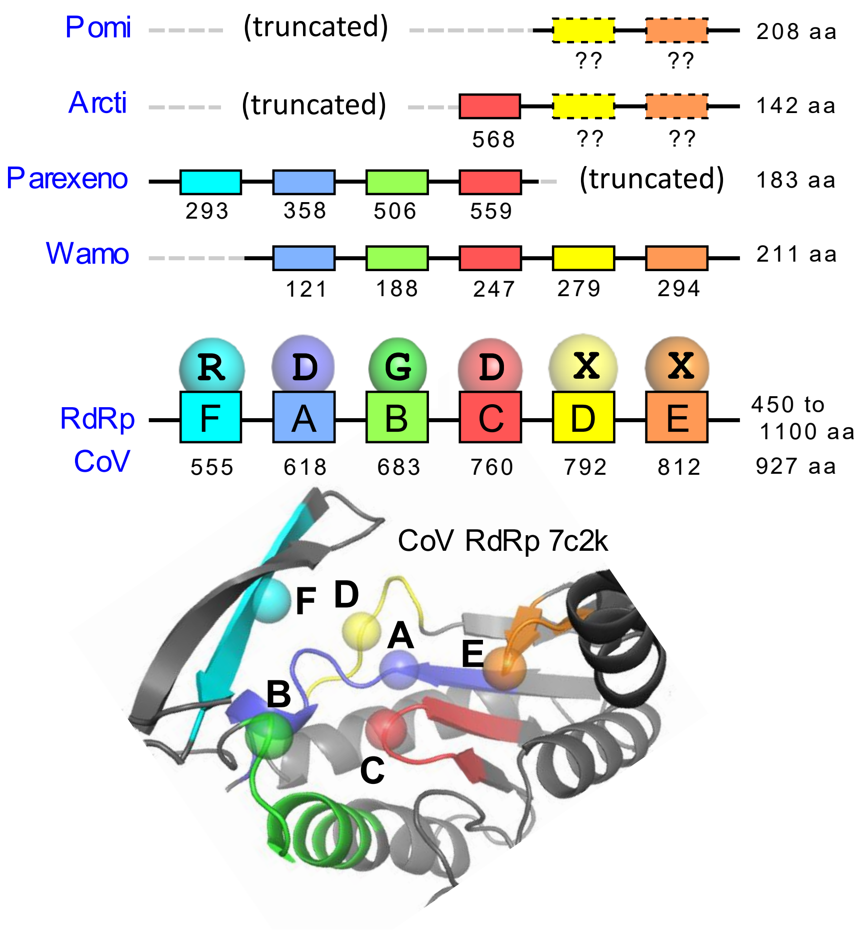

Fig. SN6.2. Structural alignments of Tara predicted structures to a solved viral RdRp. Four of Tara's predicted structures are aligned to SARS-CoV-2 RdRp (PDB:72ck). The CoV structure cartoon is coloured to show the six essential motifs FABCDE, with catalytic residues indicated by spheres. Above the cartoon is a schematic showing inferred motif positions in each structure as residue numbers in the pdb files (note that residue numbers are not 1-based). Chain lengths are given at the right-hand side. Phylum names are abbreviated to Pomi="Pomiviricota", Arcti="Arctiviricota" etc. All Tara structures except "Taraviricota" (not shown) are truncated such that one or more motifs are missing. Motifs D and E in solved structures did not align well to the corresponding regions in the highly truncated predicted Pomi and Arcti structures, and I was therefore not able to verify the presence of these motifs (see Fig. SN6.3).

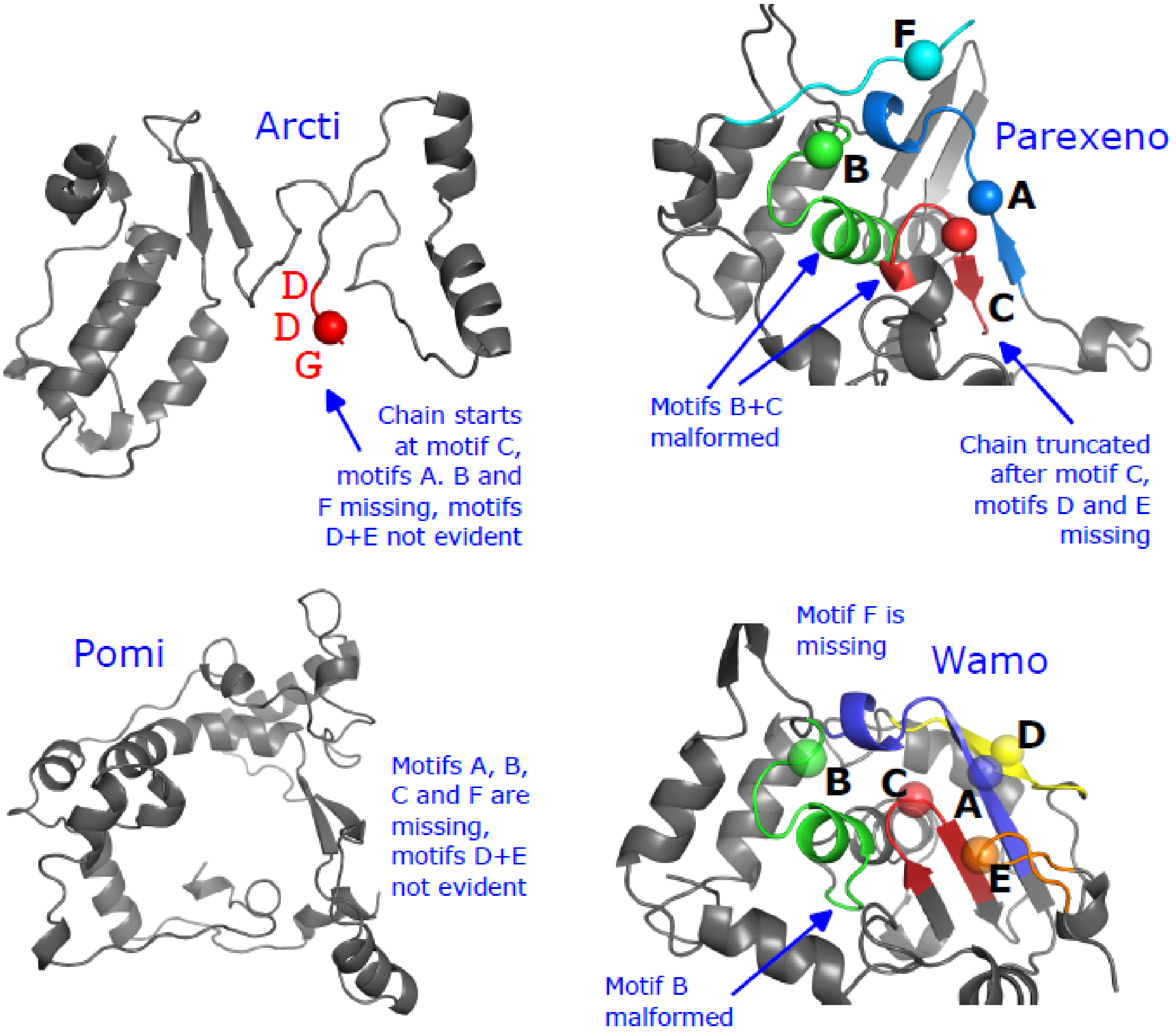

Fig. SN6.3. Truncated and malformed palm domains in predicted structures.
The figure shows the complete structures of Arcti, Parexeno, Pomi and Wamo as archived in Tara's cyverse repository (phylum names without "viricota" for brevity). None of these structures has a complete palm domain. The Pomi structure is truncated such that the entire chain aligns approximately to a region starting after motif E, which implies that all conserved motifs are deleted if in fact they are present. In Arcti, the first three residues of the chain are GLY-ASP-ASP which align approximately to motif C, but the characteristic conformations of motifs C, D and E are not evident. Parexeno and Wamo align to roughly half of the palm domain, but the catalytic core is obviously malformed (see figure in main text).

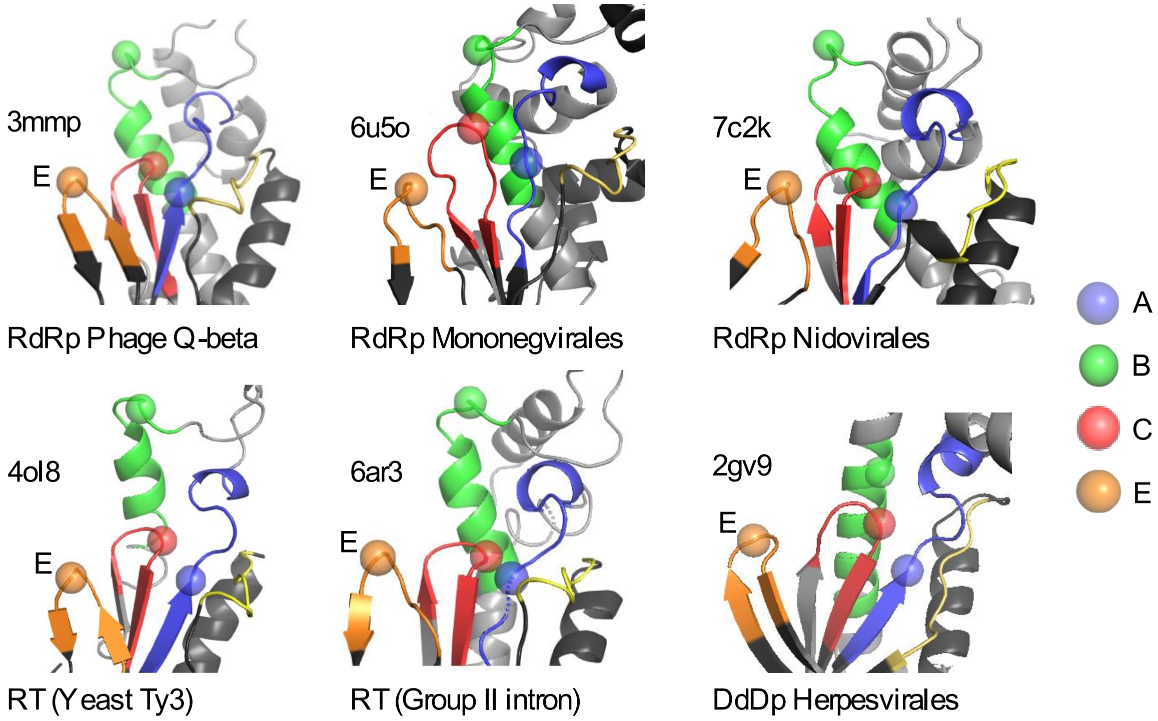

Fig. SN6.4. Conservation of motif E in the palm domain superfamily.
The figure shows the catalytic core of the palm domain in three viral RdRp structures (top) together with two cellular reverse transcriptases and a DNA-dependent DNA polymerase from *Herpesvirales*. Motif E is found in all solved viral RdRps, exhibiting a characteristic antiparallel beta sheet conformation similar to motif C. Motifs C and E are parallel to each other with their U-turns appearing on the exposed surface of the palm itself, where the tops of the U-turns form a well-conserved triangle with the catalytic ASP in motif A. Here, the structure cartoons are rotated to place motif E in the foreground to make these features easily visible. Motif E is located at residue 397 in 3mmp:G (*Lenarviricota*), contradicting the claim in Tara's Fig. S5 that motif E is "naturally absent" in this structure (see also Fig. SN6.5).

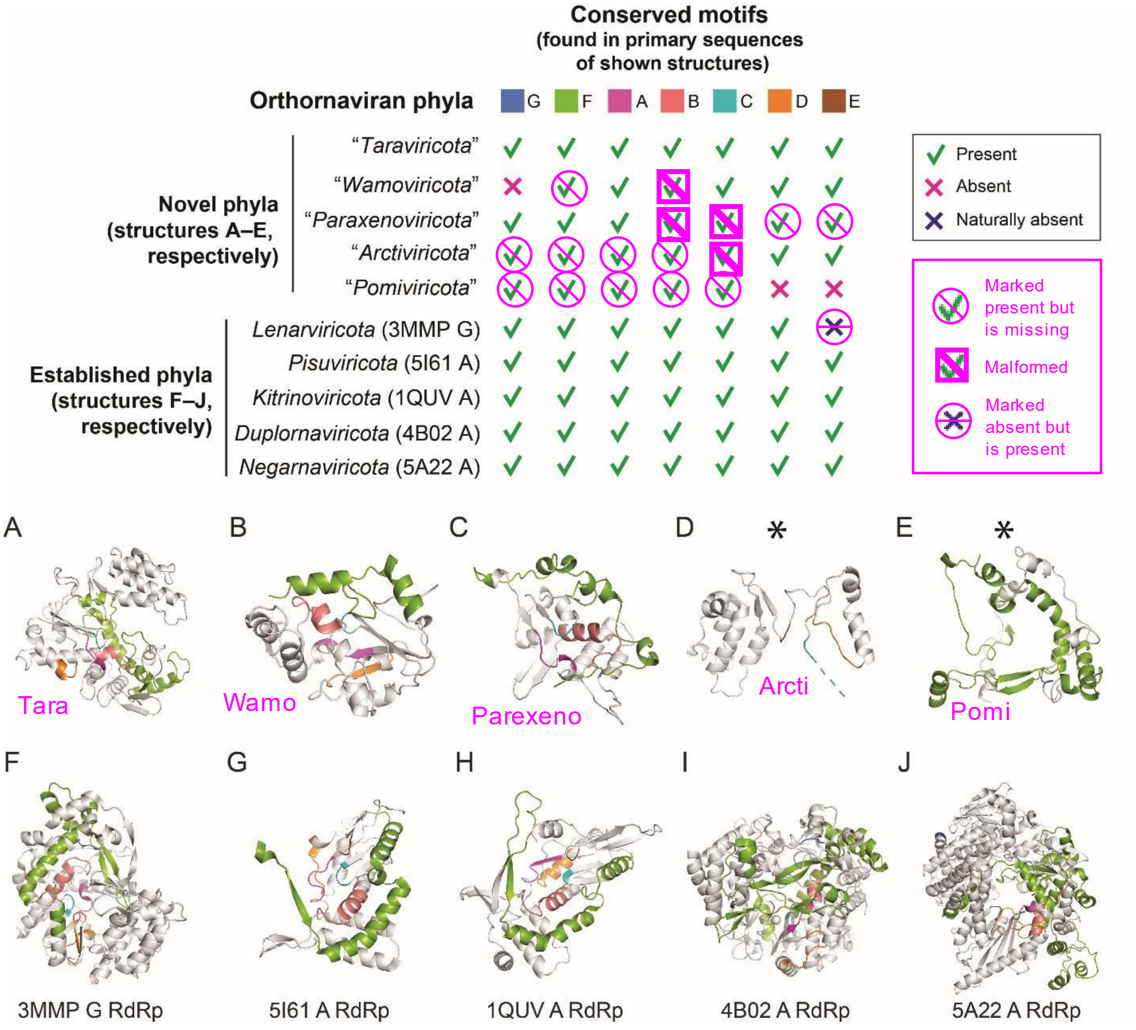

Fig. SN6.5. Incorrect motif identifications in Tara Supp. Fig. S5.
The figure shows Tara's supplementary Fig. S5, with my notes added (magenta colour). I find 12 motifs marked as present by Tara to be definitively absent due to truncation of the domains. Four motifs are present but obviously malformed. Essential motif E is annotated as "naturally absent" in PDB:3mmp by Tara, but in fact this motif is present at residue 397 as shown above in Fig. SN6.4.

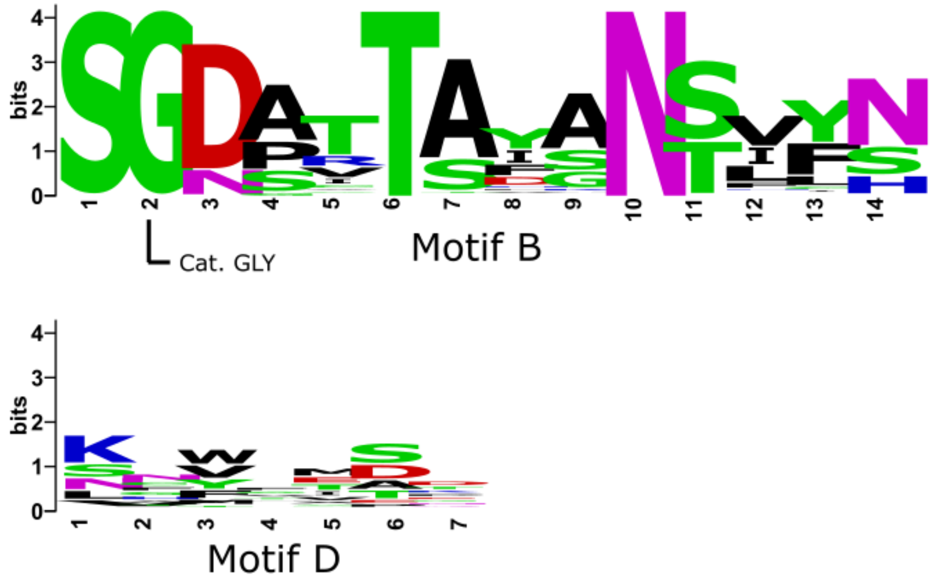

Fig. SN6.6. Sequence conservation of motifs B and D in *Nidovirales*.
Sequence logos by weblogo (Crooks et al. 2004) from a Muscle5 alignment. As seen here, even within a single order motif D is so weakly conserved as to be effectively unrecognizable, while motif B is highly conserved in sequence and readily recognized visually or by a PSSM. Between phyla, all motifs are challenging to recognize by sequence, as shown by the Wolf2018 misalignments of A, B and C reported in the Muscle5 paper (Edgar 2022).
