# Supplementary material for: Known phyla dominate the Tara Oceans RNA virome
Source: Virus Evol. 2023 Nov 8;9(2):vead063. doi: 10.1093/ve/vead063 (PMC10649353; doi:10.1093/ve/vead063)
Supplement: vead063_Supp [file vead063_supp.zip › Supplementary_Note_N7_RdRp_structure_analysis_in_pymol.pdf]

Known phyla dominate the Tara Oceans RNA virome

Robert C. Edgar

Supplementary Note N7: RdRp structure analysis in pymol

In this work, I used a manual procedure for finding structural motifs, because in my experience automated structural aligners often fail to correctly align all motifs, which precluded a straightforward automated approach. With practice, I found that the motifs are readily recognizable. I am grateful to Artem Babaian for teaching me how to recognize the D, E and F motifs.

I suggest the following exercise for learning how to do this. Start by collecting PDB identifiers and their motifs from the literature; you could use Table SN7.1 as a starting point. Here Lang2012 (https://doi.org/10.1093/nar/gks1251) is an excellent resource with positions of all motifs in solved palm domain structures known in 2012 and citations to original papers where motifs were identified.

For each structure where you have motif sequences or coordinates from a trusted source, load it into pymol, select each motif in turn, and apply a distinctive colour. For my figures in this work, I used the following colours: A=tv_blue, B=tv_green, C=tv_red, D=yelloworange, E=tv_orange and F=cyan. Then, hide all atoms outside the palmcore. Strictly, the palmcore starts 150aa before motif A and ends 150aa after motif C by definition, but rough numbers are fine here; the goal is isolate the core of palm domain by hiding distracting visual clutter from the rest of the structure. Save a pymol session for each structure. Lang2012 describe 18 structures; I recommend annotating all of these as a learning exercise to get a feel for palm domain structural features, and to construct a visual reference.

Load the coloured palmcores into new sessions, and align them by manually rotating them into corresponding orientations. For each essential catalytic residue (ASP in motifs A and C, GLY in motif B), make a C-alpha selection and use the "draw sphere" command. This highlights the well-conserved positions of the essential residues within the conserved shapes of the motifs. Note how the connectors from C to D to E have similar secondary structures of roughly similar lengths. Note the position of motif F relative to the palmprint segment (A...B...C) (above the palmprint in the orientation used in my figures here). Save these aligned sessions for future reference. Make a "cheat-sheet" figure with, say, six representative coloured structures displayed

in a grid. Repeat this for, say, two or three different orientations. These figures are convenient for quick visual comparison with a new structure.

To annotate a new structure not described in the literature, e.g. a prediction by an AI folding algorithm, I found the easiest starting point is usually to use palmscan to locate the A, B and C motifs. The "select pepseq" command of pymol is handy for quickly locating and selecting motif sequences reported by palmscan. Otherwise, you can look for the distinctive shape of the C motif (anti-parallel beta sheet with U-turn, with ASP residue in the U-turn) and/or its characteristic GLY-ASP-ASP sequence. Once you have found motif C, motifs A and B should be easily recognizable from their shapes and positions relative to C. Colour A, B and C using the same convention as your reference set, and rotate the new structure into one of your reference orientations for comparison. The locations of the remaining D, E and F motifs should then be apparent by comparison with the references. An alternative approach is to use the pymol "align" command to align the new structure to one or more of your annotated references; here some trial and error will be needed to find the best match. If a good structural alignment is obtained, then approximate motif annotations should be easily transferred; adjustment by a few residues may be needed to correctly position the catalytic motifs.

Reference

Lang, D. M., Zemla, A. T., and Zhou, C. L. E. (2012). Highly similar structural frames link the template tunnel and NTP entry tunnel to the exterior surface in RNA-dependent RNA polymerases. Nucleic Acids Research, 41(3):1464–1482.

PDB	A	B	C	D	E	F	
**7c2k:A**	MGWDYPKCDRAM	SGDATTAYANSVFN	ILSDDAVV	NNVFMSE	HEFCSQH	RARTVAG	**RdRp**
**2cjq:A**	VSFDTKNWDTQV	SGQPDTSAGNSMLN	VCGDDGFL	KPQKITE	IEFCSHT	NPRVIQY	**RdRp**
**1nb7:A**	FSYDTRCFDSTV	SGVLTTSCGNTLTC	VNGDDLVV	APPGDPP	ITSCSSN	PARLIVF	**RdRp**
**1hhs:A**	VATDVSDHDTFW	SGQGATDLMGTLLM	SKSDDAML	MKISYEH	GAFLGDI	RRRTAMG	**RdRp**
**7c2k:A**	MGWDYPKCDRAM	SGDATTAYANSVFN	ILSDDAVV	NNVFMSE	HEFCSQH	RARTVAG	**RdRp**
**6ar3:A**	VDMDLEKFFDRV	QGGPLSPLLANILL	RYADDCNI	LKVNEEK	RAFLGFS	GTRQLGI	**G2RT**

Table SN7.1. Motifs in representative palm domain superfamily structures. Annotated by visual inspection.
